# HnRNP D activates production of HPV16 E1 and E6 mRNAs by promoting intron retention

**DOI:** 10.1093/nar/gkac132

**Published:** 2022-03-02

**Authors:** Xiaoxu Cui, Chengyu Hao, Lijing Gong, Naoko Kajitani, Stefan Schwartz

**Affiliations:** Department of Laboratory Medicine, Lund University, BMC-B13, 221 84 Lund, Sweden; Department of Laboratory Medicine, Lund University, BMC-B13, 221 84 Lund, Sweden; Department of Laboratory Medicine, Lund University, BMC-B13, 221 84 Lund, Sweden; China Institute of Sport and Health Sciences, Beijing Sport University, Haidian District, Beijing 100084, China; Department of Laboratory Medicine, Lund University, BMC-B13, 221 84 Lund, Sweden; Department of Medical Biochemistry and Microbiology (IMBIM), Uppsala University, BMC-B9, 751 23 Uppsala, Sweden; Department of Laboratory Medicine, Lund University, BMC-B13, 221 84 Lund, Sweden; Department of Medical Biochemistry and Microbiology (IMBIM), Uppsala University, BMC-B9, 751 23 Uppsala, Sweden

## Abstract

Human papillomavirus type 16 (HPV16) E1 and E6 proteins are produced from mRNAs with retained introns, but it has been unclear how these mRNAs are generated. Here, we report that hnRNP D act as a splicing inhibitor of HPV16 E1/E2- and E6/E7-mRNAs thereby generating intron-containing E1- and E6-mRNAs, respectively. N- and C-termini of hnRNP D contributed to HPV16 mRNA splicing control differently. HnRNP D interacted with the components of splicing machinery and with HPV16 RNA to exert its inhibitory function. As a result, the cytoplasmic levels of intron-retained HPV16 mRNAs were increased in the presence of hnRNP D. Association of hnRNP D with HPV16 mRNAs in the cytoplasm was observed, and this may correlate with unexpected inhibition of HPV16 E1- and E6-mRNA translation. Notably, hnRNP D40 interacted with HPV16 mRNAs in an HPV16-driven tonsillar cancer cell line and in HPV16-immortalized human keratinocytes. Furthermore, knockdown of hnRNP D in HPV16-driven cervical cancer cells enhanced production of the HPV16 E7 oncoprotein. Our results suggest that hnRNP D plays significant roles in the regulation of HPV gene expression and HPV-associated cancer development.

## INTRODUCTION

Human papillomaviruses (HPV) contain a double-stranded circular DNA genome of approximately 8 kb in size (1). HPV infections cause a range of disease from benign warts to invasive cancers, for example cervical cancer and tonsillar cancer (2). HPV type 16 (HPV16) is responsible for around 55% of all cervical cancers while the remainder is caused by other high risk (HR) HPV types (3). Cancer progression is due to an increased continuous expression of HPV oncoproteins E6 and E7 that inactivate tumor suppressor proteins p53 and pRb (4), respectively. E6 and E7 activate the cell cycle, inhibit apoptosis and cause genomic instability (5–9). The HPV16 E1 and E2 proteins are key factors during replication of HPV16 genomic DNA. E1 functions as DNA helicase whereas E2 has a multifunctional role including transcriptional regulation, initiation of HPV16 DNA replication, facilitation of HPV16 genome partitioning during mitosis and post-transcriptional control of HPV16 gene expression (10–13). In contrast to E6 and E7, E2 has pro-apoptotic properties and is frequently inactivated when the HPV16 genome integrates in cellular chromosomes, a process that possibly enhances carcinogenesis (11,14). The HPV16 E4 and E5 proteins are essential for completion of the HPV16 replication cycle and E5 may contribute to carcinogenesis (15,16).

Since the HPV16 genome has two promoters only, alternative mRNA splicing plays a major role in the regulated expression of all HPV16 genes (17–22). A complex pattern of alternatively spliced and polyadenylated HPV16 mRNAs is observed during the HPV16 life cycle. Therefore, it is not surprising that a number of cis-acting regulatory RNA elements and their cognate trans-acting factors control the HPV16 alternative splicing and polyadenylation. In addition, it has been shown that the levels of various RNA-binding proteins are altered during the progression of HPV16-infected cells to cervical cancer through a series of premalignant cervical intraepithelial lesions (23,24). Thus, it is of interest to identify cellular RNA-binding proteins that control HPV16 gene expression.

Heterogeneous nuclear ribonucleoproteins (hnRNPs) represent a large family of RNA-binding proteins (RBPs). The association of hnRNP proteins with pre-mRNAs is initiated co-transcriptionally at the nascent transcripts. Many of the RNA-binding proteins remain bound to the resulting mRNAs all the way to the ribosomes and shuttle back and forth between the nucleus and the cytoplasm, demonstrating that RNA-binding proteins are important determinants of pre-mRNA processing during the entire mRNA pathway including mRNA splicing, localization, translation and stability (25). hnRNPs are massively involved in alternative splicing and the canonical function of hnRNPs is performed through their binding to RNA elements adjacent to splice sites, thereby either repressing or supporting the assembly of the spliceosome complex on splice donor (SD) or splice acceptor (SA) sites. Alternatively, they affect the recruitment of other RNA-binding proteins such as Serine/Arginine (SR) rich proteins to exonic or intronic splicing enhancers or silencers (26). Furthermore, many hnRNPs participate in more than one of these processes (27), e.g. regulation of both alternative splicing- and poly(A)-site usage by hnRNP H/F or L, alternative splicing and translation by hnRNP A1, or alternative splicing and RNA stability by hnRNP D.

The hnRNP family comprises at least 20 major RNA-binding proteins originally named alphabetically from A1 to U. These proteins share modular structures that include RNA recognition motifs (RRM) or quasi-RRMs, other motifs such as the K Homology (KH) domain and the arginine/glycine-rich RGG motif and other RNA-binding domains (RBD) present on a subset of hnRNPs (28). In some hnRNPs other auxiliary domains like glycine rich domains and proline-rich domains may be present (25,29). Although all hnRNPs display RNA-binding activity with a certain degree of specificity, an hnRNP protein may not bind exclusively to high-affinity binding sites. The RNA-binding specificity of hnRNPs is strongly influenced by the type and number of RNA-binding domains on the hnRNP, which in turn generates both general and specific interactions with nucleic acids, as well as the primary and secondary structures of the target RNA (30). HnRNPs also possess nuclear localization sequences (NLSs) that bring the proteins to the nucleus, while other hnRNPs possess nucleocytoplasmic shuttling domains which allow hnRNPs to shuttle between nuclear and cytoplasmic compartments (28). Given the important role of hnRNPs in mRNA metabolism, we speculate that HPV16 early gene expression is under control of a range of hnRNPs. Here we determined the effect of 13 different hnRNPs on HPV16 early mRNA splicing and we found that a number of the hnRNPs affected HPV16 early mRNA splicing, but in different ways. We focused our attention on the members of the cellular hnRNP D protein family. hnRNP D, also known as AU-rich element binding factor 1 (AUF1) promotes the decay of many target mRNAs (31), but it was also reported to enhance the stability and affect translation of target transcripts. As a result, hnRNP D is involved in multiple cellular processes, including miRNA biogenesis (32), translational regulation (33,34), telomere maintenance (35,36), cell cycle control (37), apoptosis (38) and inflammatory responses (39) since hnRNP D target mRNAs encode proteins implicated in these processes. Reports on hnRNP D and mRNA splicing are relatively scarce but a regulatory function of hnRNP D proteins in alternative splicing has been suggested (34). One example is that hnRNP D proteins as well as hnRNP DL control their own expression by auto- or cross-alternative splicing regulation (40). Another example suggests that hnRNP D together with neuronal members of the ELAVL protein family (nELAVLs) induce neuron-specific alternative splicing of the amyloid precursor protein (APP) (41).

In this manuscript, we show that hnRNP D proteins inhibit splicing and promote retention of the E1- and E6-encoding introns on the HPV16 early mRNAs, thereby specifically stimulating production of the partially spliced HPV16 E1- and E6-encoding mRNAs. Furthermore, hnRNP D facilitated export of the partially spliced HPV16 E1 and E6 mRNAs to the cytoplasm. However, despite the fact that the HPV16 E1 and E6 mRNAs reached the cytoplasm in association with hnRNP D, HPV16 mRNAs were poorly translated. In conclusion, our results suggest that hnRNP D proteins inhibit HPV16 early mRNA splicing and enhance production of the partially spliced, intron-containing HPV16 E1 and E6 mRNAs, thereby playing an important role at the initial steps of the biogenesis of HPV16 E1 and E6 mRNA.

## MATERIALS AND METHODS

### Cells

HeLa, 293T, SiHa and C33A2 cells were cultured in Dulbecco’s modified Eagle medium (DMEM) (HyClone) with 10% bovine calf serum (HyClone) and penicillin/streptomycin (Gibco). C33A2 cell line has been described previously (42). Briefly, C33A2 is an in-house cell line derived from C33A cells stably transfected with HPV16 reporter plasmid pBELsLuc (43). HPV16-infected tonsillar cancer cell line HN26 has been described previously (44). Briefly, the HN26 cells are derived from a tumor of a 48-year-old nonsmoking man with non-keratinizing, HPV16-positive tonsil oral squamous cell carcinoma, stage T2N0M0. The HN26 cells contain episomal HPV16 DNA and have an intact p53 gene. HN26 cells were cultured in RPMI 1640 medium (HyClone) with 10% iron-supplemented bovine calf serum (HyClone), 5% MEM non-essential amino acid solution (Sigma Aldrich) and 5% sodium pyruvate (Sigma Aldrich). Neonatal Primary Normal Human Foreskin Keratinocytes (nHFK, purchaced from Themo Fisher Scientific) and 3310 cells were cultured in EpiLife medium (Gibco) supplemented with 1% human keratinocyte growth supplement (HKGS, Gibco) and 0.2% Gentamicin/Amphotericin (Gibco). Differentiation of nHFK was induced by addition of CaCl_2_ at a final concentration of 2.4 mM in the keratinocyte culture medium for 24 h. The HPV16-immortalized keratinocyte 3310 cell line has been described previously and was generated by stable transfection of nHFK with HPV16 genome plasmid pHPV16ANE2fs (43).

### Plasmids

The following plasmids have been described previously: pHPV16AN (43), pC97ELsluc (42) and pBELsluc (43). Construction of phnRNP F, phnRNP I, phnRNP A2 and phnRNP Q has been described previously (45) and so has phnRNP A1 (46) and phnRNP C1 (47). Plasmids Flag-p37, Flag-p40, Flag-p42 and Flag-p45 were kindly given by Dr R. J. Schneider (48), hnRNP G plasmid by Dr I. C. Eperon (49), histidine and myc-tagged hnRNP L plasmid by Dr S. Guang (50) and histidine and myc-tagged hnRNP R plasmid by Dr P. Xu (51). phnRNP AB encoding myc-tagged hnRNP AB transcript variant 1 (RC204360) and phnRNP DL encoding hnRNP DL transcript variant 2 (RC204064) were purchased from OriGene Technologies. phnRNP H contains the hnRNP H open reading frame driven by a CMV promoter. It was constructed by PCR-amplification of hnRNP H coding sequence from pET-15B-hnRNP H (generously provided by Dr D. Black) (52) followed by subcloning into pCL086 (53).

To construct HPV16 subgenomic plasmids pX656 and pX478, primer B97S (Supplementary Table S1) was used in combination with primer X656A or X478A to amplify HPV16 sequences that were digested with PteI and XhoI and inserted between the PteI and XhoI sites in CMV promoter driven empty vector pCL086 (53). HPV16 subgenomic plasmid pXH856F encoded HPV16 E6 and E7 genes (HPV16 nucleotides positions 104-855) (HPV16 nucleotide positions refer to the HPV16 reference sequence HPV16R (GeneBank: K02718.1)). E6 was fused to an HA tag sequence at the 5′-end and E7 to a flag tag sequence at the 3′-end. pXH856F was generated by insertion of DNA fragment PCR-amplified with pXH856F sense and anti-sense primer (Supplementary Table S1) and subcloned into pCL086 at PteI and XhoI sites. The 5′-splice site SD226 in pXH856F was mutated (T228C, A229C) creating pXH856SDmF. The mutations in SD226 were introduced by PCR mutagenesis with pXH856SDmF sense and antisense primers (Supplementary Table S1) in a two-step PCR amplification reaction using overlapping followed by subcloning into pXH856F. Plasmid p16E1-3xF encodes the HPV16 E1 gene (HPV16 nucleotides positions 865-2811) fused with a 3xFLAG tag sequence at the 3′-end and was constructed by PCR amplification using p16E1-3xF inverse sense and anti-sense primers (Supplementary Table S1) and subcloned into pCL086. HPV16 5′-splice sites SD880 and SD1302 on p16E1SDm-3xF were mutated (G881C and G1303C) to create p16E1SDm-3xF. The mutations were conducted by p16E1SDm-3xF sense and antisense primers (Supplementary Table S1) in two-step PCR amplification.

To construct HPV subgenomic plasmids pBELEN, pBELENdE1, pBELsluc plasmid was cut with restriction enzymes CsiI and XhoI, followed by blunting of sticky DNA ends with Klenow fragment and re-ligation, resulting in pBELEN. To construct pBELENdE1, pBELEN was cut with restriction enzymes AdeI and NsiI to delete sequences in the E1 coding region, followed by blunting of sticky DNA ends with Klenow fragment and re-ligation.

To construct plasmid pFLAG-hnRNPD40, the hnRNPD40 sequence was fused to a FLAG-tag sequence at the 5′-end by PCR amplification with primers pflag-D40 sense and anti-sense (Supplementary Table S1) using plasmid FLAG-p40 (48) as template. To construct hnRNPD40 deletion mutants pD1, pD2 and pD5, hnRNPD40 sequences fused to flag tag at the 5′-end were first PCR-amplified with primers ‘pD1-, pD2- or pD5-sense’ and ‘pflag-D40 anti-sense’ (Supplementary Table S1) from plasmid pflag-hnRNPD40. To construct hnRNPD40 deletion mutants pD3, pD4 and pD7, hnRNPD40 sequences fused to a flag tag at the 5′-end were first PCR-amplified with primers ‘pflag-D40 sense’ and ‘pD3-, pD4- or pD7- anti-sense’ (Supplementary Table S1) from plasmid pFLAG-hnRNPD40. To construct hnRNPD40 deletion mutant pD8, hnRNPD40 sequences fused to a FLAG tag at the 5′-end were first PCR-amplified with primers ‘pD1 sense’ and ‘pD4 anti-sense’ (Supplementary Table S1) from plasmid pFLAG-hnRNPD40. The PCR-fragments mentioned above were digested with restriction enzymes PteI and XhoI and subcloned into plasmid pCL086 cut with PteI and XhoI. pD9 was constructed by blunt end re-ligation of pFLAG-hnRNPD40 cut by StuI and XhoI. pflag-hnRNPD40 and deletion mutants are shown schematically in Figure 4A. To construct hnRNPD40 substitution mutants pQ6A and pAGG, hnRNP D40 sequences (BglII and XhoI) encoding hnRNPD40 mutant fragments were synthesized by Eurofins Genomics. These fragments were digested with restriction enzymes BglII and XhoI and subcloned into plasmid pFLAG-hnRNPD40 digested with BglII and XhoI. To construct pD1 and pD2 substitution mutants pD1-AGG, pD1-Q6A, pD2-AGG and pD2-Q6A, plasmids pAGG or pQ6A were digested with BglII and XhoI and subcloned into plasmid pD1 and pD2 digested with BglII and XhoI. Substitution mutants are shown schematically in Figure 5A. To generate hnRNP D40-plasmids fused to GFP, primer ‘pEGFP-D40 sense’ was first used in combination with primer ‘pEGFP-D40-, pEGFP-D3-, pEGFP-D4-, pEGFP-D7- or pEGFP-D9-anti-sense’ (Supplementary Table S1) to amplify full-length hnRNPD40 or deletion mutant sequences of hnRNPD40 from pflag-hnRNPD40, pD1, pD2, pD5, pD3, pD4, pD8, pD7 or pD9, respectively. The PCR-fragments were digested with HindIII and BamHI and inserted between the HindIII and BamHI sites in the pEGFP-C3 vector (Clontech) resulting in pEGFP-D40, pEGFP-D1, pEGFP-D2, pEGFP-D5, pEGFP-D3, pEGFP-D4, pEGFP-D8, pEGFP-D7 and pEGFP-D9.

To construct the E6 protein encoding plasmid with T7 promotor pET32a-HA-E6SDm, E6 sequences were PCR amplified from pXH856SDmF using pET32a-HA-E6SDm sense and antisense primers (Supplementary Table S1) followed by subcloning into pET32a using EcoRI and XhoI sites. To construct the E1 protein encoding plasmid with T7 promotor pcDNA3.1(+)-E1SDm-3XFlag, E1 sequences were released from plasmid p16E1SDm-3xF by HindIII and XhoI digestion followed by subcloning into pcDNA3.1(+) digested with HindIII and XhoI.

### Transfections

Transfections of HeLa cells and 293T cells were performed with Turbofect according to the manufacturer’s protocol (Thermo Fisher Scientific). Briefly, a mixture of Turbofect:DNA ratio of 2:1 (μl reagent: μg DNA) for HeLa cells or 4:1 for 293T cells and DMEM without serum was incubated at room temperature for 20 min prior to dropwise addition to subconfluent cells. Transfections of nHFK were performed with ViaFect according to the manufacturer’s protocol (Promega). In brief, a mixture of ViaFect:DNA ratio of 3:1 and EpiLife without serum was incubated at room temperature for 20 min prior to dropwise addition to subconfluent cells. Fluorescence images of EGFP set of plasmids transfected HeLa cells were acquired using Olympus CKX53 inverted microscope.

### Nuclear and cytoplasmic extraction

Nuclear and cytoplasmic extracts were prepared from HeLa cells grown in 6 cm dishes at 24 h post-transfection. Cells were harvested by using NE-PER Nuclear and Cytoplasmic Extraction Reagents (Thermo Fisher Scientific) according to the manufacturer’s protocol. In brief, cell pellets were resuspended in ice cold buffer CER I with protease inhibitors (Sigma Aldrich) and vortexed vigorously prior to incubation on ice for 10 min. Ice-cold buffer CER II was added to the samples that were vortexed vigorously and incubated on ice for one minute. After 5 min of maximum speed centrifugation, the supernatants were collected as cytoplasmic extracts. The pellets were washed once by PBS and collected as nuclear extracts.

### RNA extraction and RT-PCR

Total RNA was extracted from transfected cells using TRI Reagent (Sigma Aldrich) and Direct-zol RNA MiniPrep (ZYMO Research) according to the manufacturer’s protocols. Reverse transcription (RT) was performed in a 20 μl reaction using random hexamers (Invitrogen) and reverse transcriptase (Invitrogen). One microliter of cDNA was subjected to PCR-amplification. cDNA representing HPV16 mRNAs spliced from HPV16 5′-splice site SD226 to 3′-splice site SA409 was amplified with RT-PCR primers 97S and 438AS (Supplementary Table S1) and cDNA representing HPV16 mRNAs spliced from 5′-splice site SD226 to 3′-splice sites SA409, SA526, or SA742 with RT-PCR primers 97S and 880AS (Supplementary Table S1). cDNA representing HPV16 mRNAs spliced from 5′-splice site SD880 to 3′-splice site SA2709 or SA3358 was amplified with RT-PCR primers 773S and E2AS/E2QAS or E4AS (Supplementary Table S1). cDNA representing HPV16 mRNAs spliced from 5′-splice site SD226 to 3′-splice site SA2709 or SA3358 were amplified with RT-PCR primers 97S and E2AS/E2QAS or E4AS, respectively (Supplementary Table S1). Glyceraldehyde-3-phosphate dehydrogenase (GAPDH) cDNA was amplified with primers GAPDHF and GAPDHR (Supplementary Table S1). Spliced actin and unspliced actin cDNAs were amplified with primers actin-s or actin-s1 and actin-a (Supplementary Table S1). cDNA representing mRNAs encoding each of the four isoforms of hnRNP D were amplified with hnRNP D mRNA-specific primers 2S and 7A (Supplementary Table S1). For location of these mRNAs see Figure 3A. To monitor recombination at the loxP sites in pHPV16AN, PCR was performed with primers 16S and 16A on DNA extracted from the transfected cells (this PCR yields a 366-nucleotide PCR fragment that is diagnostic for recombination at the LoxP sites). Examples of control PCR experiments performed on RNA samples in the absence of reverse transcriptase are shown in various figures. Primer pairs used for RT-PCR and RT-qPCR (described in next section) are summarized in Supplementary Table S4.

### Real-time quantitative PCR (qPCR)

**Figure 1.**
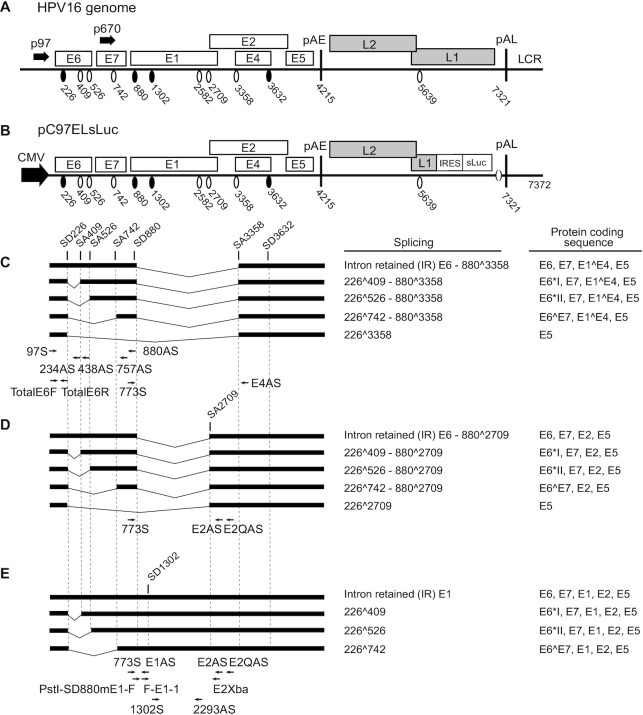
Schematic representation of the HPV16 genome, HPV16 subgenomic plasmid pC97ELsLuc and representative HPV16 mRNAs. (**A**) Linearized HPV16 genome (numbers refer to the HPV16 reference strain GeneBank: K02718.1). Early and late genes are indicated. P97: HPV16 early promoter. P670: HPV16 late promoter. Black oval: splice donor. White oval: splice acceptor. pAE: HPV16 early polyadenylation site. pAL: HPV16 late polyadenylation site. LCR: HPV16 long control region. (**B**) HPV16 subgenomic plasmid pC97ELsLuc encodes all HPV16 genes. HPV16 early promoter p97 was replaced by human cytomegalovirus immediate early promoter (CMV). Secreted luciferase (sLuc) gene was integrated in the L1 gene following the poliovirus 2A internal ribosomal entry site (IRES) sequence. (**C–E**) Schematic structures of HPV16 early transcripts produced from pC97ELsLuc. Splicing at SD226 occurs independently of splicing at the downstream SD880 and generates splice variants in the E6E7-coding region concomitantly with alternative splicing from SD880, i.e. E4 (880∧3358) (C), E2 (880∧2709) (D) or mRNAs unspliced at SD880 (E). The protein coding sequences present on each mRNA are indicated to the right. Arrows: Annealing positions of HPV16 RT-PCR primers. Primer sequences are available in Supplementary Table S1.

qPCR was performed in a final reaction volume of 20 μl with 1 μl of cDNA prepared as described above in a MiniOpticon (Bio-Rad) using the SsoAdvanced SYBR Green Supermix (Bio-Rad) according to the manufacturer’s instructions. To quantitate intron retained E6 mRNAs by RT-qPCR primers TotalE6F and 234AS were used (Supplementary Table S1). For primer location, see Figure 1C and Supplementary Figure S5. Spliced E2 mRNAs were quantitated by RT-qPCR using primers 773S and E2AS (Supplementary Table S1, for primer location, see Figure 1). For quantitation of E1 mRNA by RT-qPCR, primers 773S and E1AS were used (Supplementary Table S1, for primer location, see Figure 1). The expression levels of the mRNAs were determined from the threshold cycle (*C*t), and the relative expression levels were calculated using the 2∧ΔΔCt method. Results were normalized to GAPDH mRNA levels determined with primers GAPDHF and GAPDHR (Supplementary Table S1). mRNA quantification was performed in triplicates, and negative controls were included in each reaction. Melting curves were analyzed in each reaction.

### Secreted luciferase assay

The *Metridia longa* secreted luciferase (sLuc) activity in the cell culture medium of transfected cells was monitored with the help of the Ready-To-Glow Secreted Luciferase Reporter assay according to the instructions of the manufacturer (Clontech) as described previously (43). In brief, 50 μl of cell culture medium was added to 5 μl of 0.5X Secreted Luciferase substrate/Reaction buffer in a 96-well plate and luminescence was determined in a Tristar LB941 Luminometer.

### Western blotting

Cell extracts for western blotting were obtained by resuspending transfected cells in radioimmunoprecipitation assay (RIPA) buffer consisting of 50 mM Tris HCl pH 7.4, 150 mM NaCl, 1% NP-40, 0.5% sodium deoxycholate, 0.1% SDS, 2 mM EDTA, 1 mM DTT and protease inhibitor (Sigma Aldrich), followed by centrifugation at full speed for 20 min and collection of the supernatants. Proteins were denatured by boiling in Laemmli buffer. After SDS-PAGE, the proteins on the gels were transferred onto nitrocellulose membranes, blocked with 5% nonfat dry milk in PBS containing 0.1% Tween 20 and stained with specific primary antibodies (Supplementary Table S2) to the indicated proteins followed by incubation with secondary antibody (Supplementary Table S2) conjugated with horseradish peroxidase and detection with chemiluminescence reagents.

### ssRNA oligo pull down

Whole cell lysates of HeLa cells were prepared using cell lysis buffer consisting of 25 mM Tris HCl pH 7.4, 150 mM NaCl, 1% NP-40, 1 mM EDTA, 1 mM DTT, 200 units of RiboLock RNase Inhibitor (Thermo Fisher Scientific), Proteinase inhibitor (Sigma Aldrich) and 5% glycerol. Biotin-labeled ssRNA oligos were purchased from Sigma Aldrich (Supplementray Table S3). DynabeadsM-280 Streptavidin magnetic beads (Invitrogen) were bound to biotin-labeled ssRNA oligonucleotides in 200 μl of binding buffer (10 mM Tris, pH 7.4, 150 mM NaCl, 2.5 mM MgCl_2_, 0.5% Triton X-100) in strips of PCR tubes incubated at RT for 20 min. To pull-down proteins, 15 μg HeLa whole cell lysate was mixed with beads bound with ssRNA oligos and incubated with rotation for 1 h at room temperature followed by washing of beads 10 times with binding buffer using DynaMag 96 Side magnetic plate (Invitrogen). Proteins were eluted by boiling of the beads in Laemmli Buffer. Samples were subjected to SDS-PAGE followed by western blot analysis with the indicated antibodies (Supplementary Table S2).

### Co-immunoprecipitation

Transfected Hela cells were lysed in cell lysis buffer as described above under ‘ssRNA oligo pull down’. For immunoprecipitation, anti-flag antibody (M2, Sigma Aldrich) or IgG was added to Dynabeads protein G and incubated with cell lysates under gentle rocking at 4°C overnight. The complexes were washed six times using cell lysis buffer and eluted by boiling in Laemmli buffer. Samples were subjected to SDS-PAGE followed by western blotting with specific primary antibodies (Supplementary Table S2).

### UV-crosslinking and immunoprecipitation (CLIP)

Transfected HeLa cells grown in 10 cm dishes were washed by ice-cold PBS followed by crosslinking twice with 0.4 J cm^−2^ UV light (254 nm) in a bio-link cross-linker (Biometra). Cytoplasmic extracts were prepared as described above. Whole cell lysates were prepared by resuspending cells in one ml of RIPA buffer and incubated on ice for 30 min with occasional vortexing to lyse cells. For immunoprecipitations, 2μg of the anti-flag antibody (M2, Sigma Aldrich) or mouse IgG was incubated at 4°C overnight in 0.5 ml of cell lysate. About 20 μl of Dynabeads Protein G (10004D, Invitrogen) and 20 μl Dynabeads Protein A (10001D, Invitrogen) were blocked with 1% BSA for 0.5 h, washed three times in RIPA buffer and then added to the antibody–protein mixtures followed by incubation for 1 h at 4°C. The beads were washed three times with buffer I (50 mM Tris HCl pH 7.4, 300 mM NaCl, 0.5% NP-40, 1 mM EDTA, 1 mM DTT), three times with buffer II (50 mM Tris HCl pH 7.4, 800 mM NaCl, 0.5% NP-40, 1 mM EDTA, 1 mM DTT) and three times with buffer III (50 mM Tris HCl pH 7.4, 800 mM NaCl, 250 mM LiCl, 0.5% NP-40, 1 mM EDTA, 1 mM DTT). RNA was eluted by phenol/chloroform extraction and ethanol-precipitated and dissolved in 20 μl of water. About 10 μl of immunoprecipitated RNA was reverse transcribed using M-MLV reverse transcriptase (Invitrogen) and random primers (Invitrogen) according to the protocol of the manufacturer. Two microliters of cDNA were subjected to PCR amplification using HPV16-sepcific primer pairs 773S and E1AS, 773S and 438AS or E6F and E6R (Supplementary Table S1) as described above.

### Ribonucleoprotein (RNP) immunoprecipitation (RIP) analysis

For immunoprecipitation of endogenous ribonucleoprotein (RNP) complexes (RIP analysis) from whole cell extracts, HN26 cells or 3310 cells were lysed in cell lysis buffer. The supernatants were incubated with anti-AUF1 antibody (Cell Signalling) or IgG (Millipore) (Supplementary Table S2) overnight at 4°C. Dynabeads Protein G (10004D, Invitrogen) + 20 μl Dynabeads Protein A (10001D, Invitrogen) were added to the antibody–protein mixtures followed by incubation for 1 h at 4°C. After washing of the beads six times using cell lysis buffer, RNA was eluted using Tri reagent and incubated with 20U of RNase-free DNase I for 1 h at 37°C and subjected to RT-PCR using HPV16-sepcific primer pairs 773S and E4AS or 97S and 438AS (Supplementary Table S1) as described above.

### siRNA library and siRNA transfections

ON-TARGETplus human hnRNP D siRNA SMARTpool consists of four siRNAs to hnRNPD (L-004079-00-0010) (Dharmacon). A scrambled negative control siRNA (D-001810-10-05) was also purchased from Dharmacon. Transfections were conducted with DharmaFECT1 (Dharmacon) according to the instructions of the manufacturer. The siRNA SMARTpool to hnRNP D or scrambled control siRNAs were transfected in duplicates into C33A2 or SiHa cells grown in 12-well plates for RNA extraction and RT-qPCR or in 6-well plates for protein extraction and western blotting. Cells were harvested at 48 h post-transfection for RNA extraction and RT-qPCR or 72 h post-transfection for protein extraction and western blotting performed as described above.

### *In vitro* translation assay

*In vitro* translation was carried out with TNT(R) Quick Coupled Transcription/Translation Systems (Promega) according to the instructions of the manufacturer. In brief, 100 ng pET32a-HA-E6SDm or 200 ng pcDNA3.1(+)-E1SDm-3XFlag plasmid was translated in the absence or presence of 1 μM recombinant hnRNP D protein (EUPROTEIN) or 1 μM BSA. The 25 μl reactions were incubated for 90 min at 30°C. The translation reactions were analyzed by western blotting as described above. The Luciferase control RNA was also translated in the absence or presence of 1 μM hnRNP D or 1 μM BSA. Luciferase activity in the translation reactions were monitored according to the instructions of the manufacturer using Tristar LB941 Luminometer. Recombinant hnRNP D and BSA were separated on SDS-PAGE followed by staining with Colloidal Blue Staining Kit (Invitrogen).

### Quantitations

The softeware used to determine band intensity in western blots and RT-PCR gels is ‘Image Lab 6.1.0’ and quantitations were performed with the software ‘Prism GraphPad 8.4.0’.

## RESULTS

### hnRNP A1, hnRNP D and hnRNP I inhibit splicing of HPV16 early mRNAs

**Figure 2.**
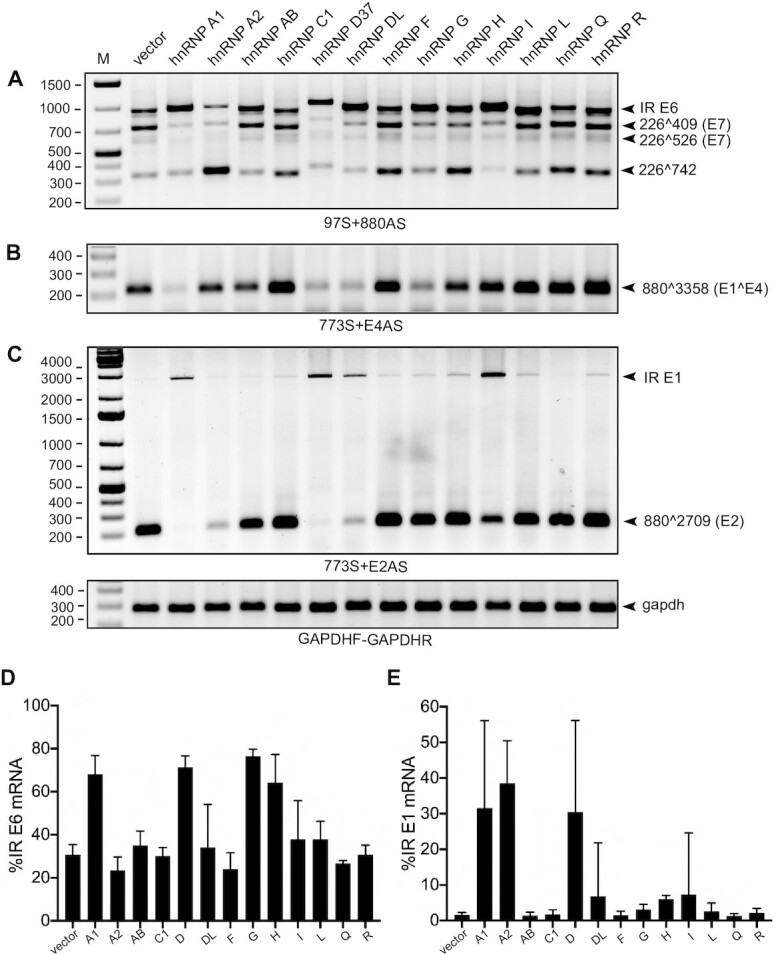
Identification of hnRNP D as a strong suppressor of HPV16 E6E7 and E1E2 mRNA splicing. (**A–C**) The effect of 13 different hnRNPs on HPV16 alternative mRNA splicing was determined by RT-PCR with different pairs of primers. HPV16 subgenomic plasmid pC97ELsLuc (Figure 1) was cotransfected individually with each of 13 different hnRNP expressing plasmid into HeLa cells. p37 isoform of hnRNP D (hnRNP D37) was used. Total RNA was extracted at 24 hpt and the various spliced HPV16 mRNAs were monitored by RT-PCR using HPV16-specific RT-PCR primer pairs: (A) E6/E7 mRNAs: 97S+880AS, (B) E4 mRNA: 773S+E4AS and (C) E1/E2 mRNAs: 773S+E2AS. Resulting RT-PCR products were electrophoresed on agarose gels and gel images are displayed. The schematic structures of the HPV16 spliced mRNAs and the locations of the HPV16-specific RT-PCR primers are indicated in Figure 1. Primer sequences are available in Supplementary Table S1. Each band was verified by sequencing and HPV16 spliced mRNA isoforms are indicated to the right of each gel. Validation of RT-PCR was also evaluated by reverse transcriptase negative samples using corresponding RNA samples. M: DNA size marker. Representative gel images of results repeated at least three-times are shown. (**D**) The effect of each hnRNP protein on the enhancement of HPV16 intron-retained (IR) E6 mRNA production shown in (A) was evaluated. A band intensity of each spliced isoform in (A) was quantitated described in Materials and Methods. The percentage of intron-retained E6 mRNA over a total sum of all four isoforms (intron-retained (IR) E6-, 226∧409-, 226∧526-, and 226∧742-mRNA) was calculated. (**E**) The effect of each hnRNP protein on the enhancement of HPV16 intron-retained E1 mRNA production shown in (C) was evaluated as described in (D).

To enhance our understanding of the regulated expression of the HPV16 early genes, we wished to identify hnRNP proteins that control splicing of HPV16 mRNAs. We therefore utilized subgenomic HPV16 reporter plasmid pC97ELsLuc that encodes all HPV16 genes and can produce all alternatively spliced HPV16 mRNAs. The schematic representation of the HPV16 genome (Figure 1A), pC97ELsLuc (Figure 1B), the structures of the HPV16 alternatively spliced mRNAs it produces (Figure 1C–E) and the location of the HPV16 RT-PCR primers are displayed in Figure 1. HPV16 plasmid pC97ELsLuc was cotransfected individually with plasmids expressing either hnRNP A1, hnRNP A2, hnRNP AB, hnRNP C1, hnRNP D, hnRNP DL, hnRNP F, hnRNP G, hnRNP H, hnRNP I, hnRNP L, hnRNP Q and hnRNP R, RNA was extracted and the various spliced HPV16 mRNAs were monitored by RT-PCR (Figure 2A–C). Quantitation of E6- and E7-encoding mRNA isoforms obtained in Figure 2A or the E1- and E2-encoding mRNA isoforms in Figure 2C are shown in Figure 2D,E, respectively. We considered three observations to be of particular interest: (i) hnRNP A1 and hnRNP A2 affected splicing of E6/E7 mRNAs as previously described with different effects on splicing of E6 and E7 mRNAs. hnRNP A1 promoted production of intron-retained E6 mRNAs, while hnRNP A2 enhanced production of 226∧742-mRNAs (46) (Figure 2A); (ii) hnRNP D, hnRNP DL, hnRNP G and hnRNP I inhibited E6/E7-mRNA splicing and enhanced production of intron-retained E6 mRNAs (Figure 2A) and (iii) hnRNP A1, hnRNP D, hnRNP DL and hnRNP I reduced the levels of spliced E2 mRNAs and promoted production of intron-retained E1-encoding mRNAs (Figure 2C,E). hnRNPs that inhibited E2 mRNA splicing also appeared to inhibit splicing between SD880 and SA3358 (E1∧E4), with the exception of hnRNP I (Figure 2B). This may be explained by the presence of a long, uninterrupted polypyrimidine tract; eleven consecutive pyrimidine upstream of E2 splice site SA2709, while the E1∧E4 splice site SA3358 has no more than four consecutive pyrimidine-nucleotides. Thus, HPV16 SA3358 would be a poor target for polypyrimidine tract binding protein/hnRNP I compared to HPV16 SA2709. All transfections have been repeated at least three times and the indicated RT-PCR products have been cut out from gels and subjected to sequencing to confirm their identities. The significant effects of hnRNP D on the majority of the HPV16 mRNAs warranted further studies on the effects of hnRNP D on HPV16 mRNA splicing.

### All four hnRNP D variants promote intron retention of HPV16 E1 and E6 mRNAs

**Figure 3.**
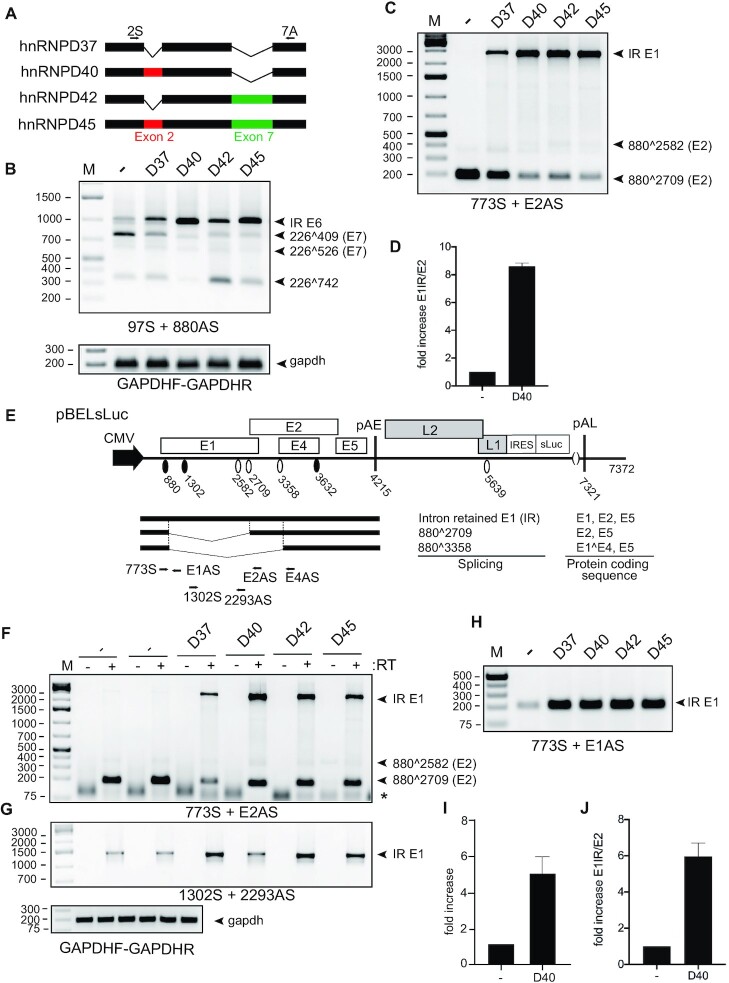
hnRNP D40 enhanced the production of intron-retained E6 and E1 mRNA more potently than the other hnRNP D isoforms. (**A**) Schematic structures of the hnRNP D protein isoforms. Each isoform differs by the inclusion of Exon 2 and/or Exon 7, respectively. Arrows: Primers’ position used for Supplementary Figure S1F. (**B** and **C**) Effect of indicated hnRNP D isoforms on HPV16 alternative mRNA splicing was investigated by HPV16 RT-PCR on RNA extracted from HeLa cells transfected with pC97ELsLuc in the absence or presence of plasmids expressing either of the hnRNP D isoforms. HPV16 RT-PCR primer pairs: (B) 97S+880AS and (C) 773S+E2AS. (**D**) RT-qPCR to quantitate intron-retained E1 mRNAs and spliced E2 mRNAs from pC97ELsLuc in the absence of presence of hnRNP D40 was performed. Fold change of intron-retained E1 mRNAs over spliced E2 mRNAs is shown. (**E**) Schematic representation of HPV16 subgenomic plasmid pBELsLuc that encodes all HPV16 genes except E6 and E7 and is driven by the CMV promoter. HPV16 mRNAs polyadenylated at pAE are shown below pBELsLuc. Arrows: Annealing positions of HPV16 RT-PCR primers. Primer sequences are available in Supplementary Table S1. (**F–H**) Effect of indicated hnRNP D isoforms on HPV16 mRNA splicing was further investigated with plasmid pBELsLuc. pBELsLuc was cotransfected with indicated hnRNP D isoform expressing plasmids into HeLa cells. Total RNA was extracted and subjected to RT-PCR using different HPV16 RT-PCR primers pairs. RT-PCR was performed in the absence (−) or presence (+) of reverse transcriptase: (F) 773S+E2AS, (G) 1302S+2293AS and (H) 773S+E1AS. Only RT (+) samples for gapdh RT-PCR are shown. (**I**) RT-qPCR with primer pairs 773S+E1AS, normalized to GAPDH levels. (**J**) RT-qPCR to quantitate intron-retained E1 mRNAs over spliced E2 mRNAs from pBELsLuc in the absence or presence of hnRNP D40. Fold change of intron-retained E1 mRNAs over spliced E2 mRNAs is shown.

We transfected plasmids expressing all four isoforms of hnRNP D (hnRNP D37, D40, D42 and D45) (Figure 3A) with HPV16 plasmid pC97ELsLuc and determined the effect on HPV16 mRNA splicing by RT-PCR (Figure 1). All four isoforms of hnRNP D are ubiquitously expressed in a variety of cell lines including normal primary human foreskin keratinocytes (nHFK) at the level of RNA (Supplementary Figure S1F). The transfection of each hnRNP D isoform expressing plasmid revealed equal levels of individual isoform protein expression with an increase of 7-fold compared to the endogeneous protein level (Supplementary Figure S1G and H). The results revealed that all hnRNP D proteins inhibited HPV16 E6/E7 mRNA splicing (Figure 3B). All hnRNP D isoforms promoted production of intron-retained mRNAs encoding E1 at the expense of the spliced E2 mRNAs (880∧2709) (Figure 3C). hnRNP D proteins also had an inhibitory effect on HPV16 E6/E7 mRNA splicing, which resulted in production of intron-retained E6-encoding mRNAs at the expense of the spliced E7 mRNAs (226∧409 and 226∧526) (Figure 3B). Furthermore, RT-PCR amplification with primer pair 97S+E1AS revealed the hnRNP D also enhanced production of an HPV16 mRNA that was intron-retained in both E6/E7- and E1/E2-regions (Supplementary Figure S1A and B), suggesting that hnRNP D affected early steps in the splicing reaction or independently inhibited splice sites in the E1- and E6-coding regions. Finally, the splicing inhibitory effect of hnRNP D on the HPV16 E6/E7 mRNAs (Supplementary Figure S1C) and the E1/E2 mRNAs (Supplementary Figure S1D), and to some extent E4 mRNAs (Supplementary Figure S1E), was dependent on the concentration of transfected hnRNP D plasmid. We concluded that hnRNP D promoted production of intron-retained HPV16 mRNAs encoding E1 or E6.

Next, we investigated if the effect of hnRNP D proteins on HPV16 intron-retained E1 mRNA production could be reproduced independently of upstream splice sites located in E6/E7 gene region. To this end, we used pBELsLuc (Figure 3E) that encodes all HPV16 genes present in pC97ElsLuc except for the E6 and the E7 genes. Since the RT-PCR products representing intron-retained E1 mRNAs (Figure 3C) could potentially originate from plasmid DNA that contaminated the RNA preparations, we performed RT-PCR in the absence or presence of reverse transcription (RT). As can be seen in Figure 3F, the bands representing intron-retained E1-encoding mRNAs were not detected in the absence of RT, nor were they easily detected in the absence of hnRNP D protein (Figure 3F). Furthermore, primers located entirely within the E1 coding region (primers 1302S+2293AS) yielded similar results as the 773S and E2AS primers regarding detection of the intron-retained E1-encoding mRNAs (Figure 3G). RT-PCR with primers 773S+E1AS (located immediately downstream of SD880 and detecting intron-retained mRNAs, Figure 3E) also showed an increase in the presence of hnRNP D (Figure 3H). It also appeared that hnRNP D40 had a stronger splicing-inhibitory effect on the HPV16 mRNAs than the remaining members of the hnRNP D family (Figure 3B–D). Thus, hnRNP D40 was used in the majority of the subsequent experiments. The effect of hnRNP D40 on intron-retained E1 mRNA production was determined by RT-qPCR with primers 773S+E1AS revealing an increase of intron-retained E1 mRNAs with 4-fold in the presence of the hnRNP D40 (Figure 3I). Furthermore, the ratio between intron-retained E1 mRNAs and spliced E2 mRNAs revealed increases of 8-fold from pC97ELsLuc (Figure 3D) and 6-fold from pBELsLuc (Figure 3J) in the presence of hnRNP D40. The enhancing effect of hnRNP D40 on production of intron-retained E1 mRNAs was further confirmed by comparisons between pC97ELsLuc and pBELsLuc (Supplementary Figure S2A and B) in the absence or presence of hnRNP D40 (Supplementary Figure S2C; 773S+E2AS) or by transfection with serially diluted hnRNP D40 plasmid (Supplementary Figure S2D). Taken together, we concluded that hnRNP D proteins inhibited splicing of HPV16 early mRNAs and promoted retention of introns encoding E1 and E6, respectively. Retention of the E1 intron occurred independently of intron retention in the E6 coding region.

### The N-terminal domain of hnRNP D40 contributes to induction of intron-retained HPV16 E1 and E6 mRNAs

**Figure 4.**
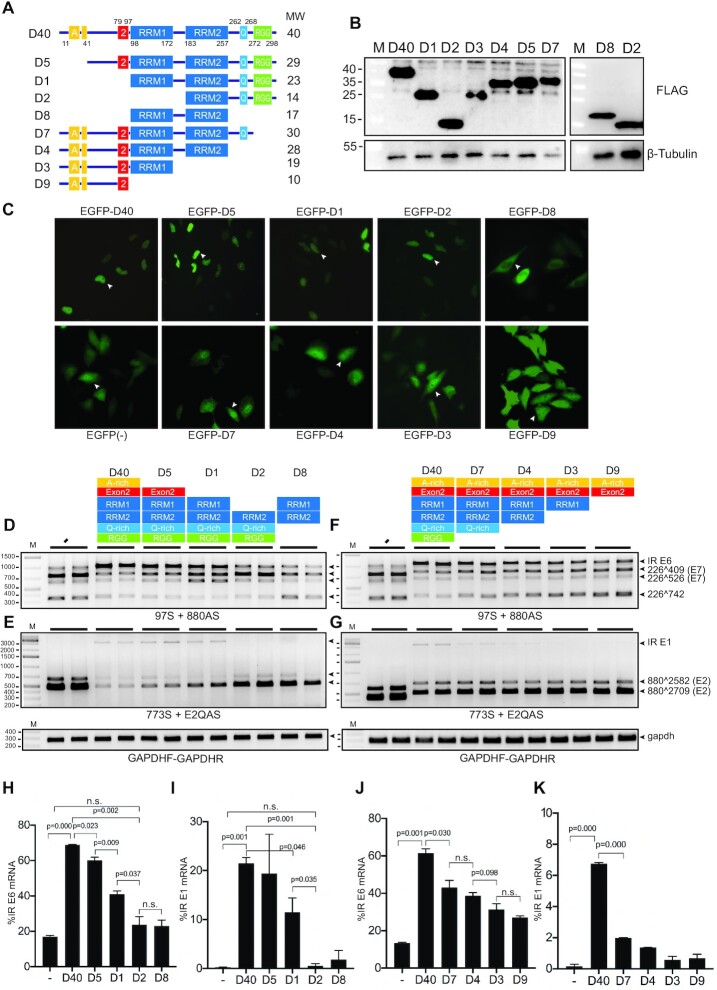
RRM1 domain of hnRNP D40 was required for HPV16 E6 and E1 intron retention whereas N-terminal and C-terminal domains contributed to modification of HPV16 early alternative mRNA splicing. (**A**) Schematic structures of wild-type hnRNP D40 and hnRNP D40 deletion mutants. All mutants were fused with FLAG-tag at its N-terminus. Box with A: Alanine(A)-rich region, box with 2: exon 2 region, box with RRM: RNA recognition motif (RRM) domain, box with Q: Glutamine(Q)-rich region and box with RGG: Arginine-Glycine/Arginine-Glycine-Glycine (RG/RGG) motif region. The names of each deletion mutant are indicated to the left and the predicted molecular weight of each protein is indicated to the right. (**B**) Western blotting against the FLAG epitope demonstrating protein expression of the hnRNP D40 deletion mutants depicted in (A). M: molecular weight marker. (**C**) Subcellular localization of indicated deletion mutants of hnRNP D40. Each mutant was fused to the C-terminus of EGFP. Representative cells expressing each mutant are highlighted by white arrowheads and shown in Supplementary Figure S3 with higher magnification. (**D–G**) Effect of FLAG-hnRNP D40 deletion mutants on HPV16 mRNA splicing was monitored by HPV16 RT-PCR on RNA extracted from HeLa cells transfected in the absence (−) or presence of hnRNP D40 or deletion mutants thereof. Representative gel images from experiments independently repeated three times are shown. (**H** and **J**) Percentage of intron-retained E6 mRNA over all four E6/E7 alternatively spliced isoforms quantified from (D) or (F). (**I** and **K**) Percentage of intron-retained E1 mRNA percentage over all three E1/E2 alternatively spliced isoforms quantified from (E) or (G). Proportions among all four isoforms is shown in Supplementary Figures S3B and S3C. Student’s *t*-test was executed and obtained *P* values were displayed; n.s., no significance.

**Figure 5.**
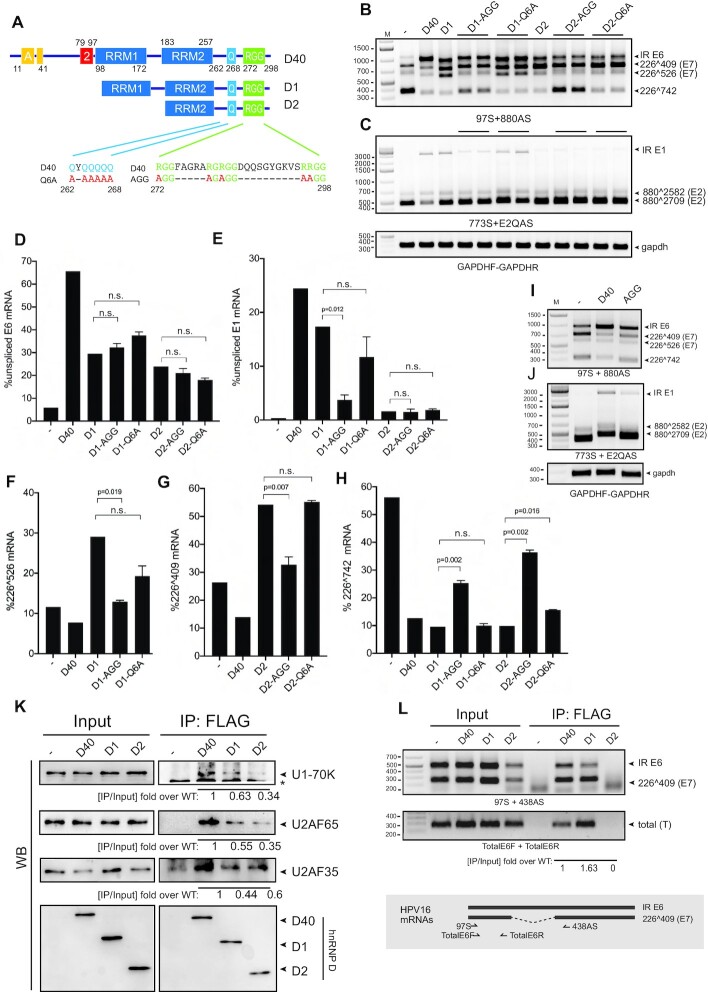
hnRNP D40 C-terminus contributes to HPV16 mRNA splicing control differently from its N-terminus. (**A**) Schematic structures of hnRNP D40 and various hnRNP D40 mutants with N-terminal deletions and C-terminal amino acid substitutions. Amino acid substitutions in the Q-rich region or the RG/RGG-motif region are indicated. Q: Glutamine, A: Alanine and R: Arginine. ‘- ’ indicates wild-type sequence. (**B** and **C**) Effect of hnRNP D40 or hnRNP D40 mutants on HPV16 E6/E7 mRNA splicing (B) or E1/E2 mRNA splicing (C) was monitored by RT-PCR with indicated primer pairs. (**D–H**) Percentage of an indicated HPV16 mRNA splice variant among all spliced isoforms of HPV16 E6/E7 or E1/E2 mRNAs was quantitated from (B) and (C) as described in Figure 2D and E: (D) percentage intron-retained E6 mRNAs, (E) percentage intron-retained E1 mRNAs, (F) percentage 226∧409 mRNAs, (G) percentage 226∧526 mRNAs and (H) percentage 226∧742 mRNAs. Student’s *t*-test was executed and obtained *P* values were displayed. n.s., no significance. (**I** and **J**) Effects of amino acid substitutions in the RG/RGG region of full-length hnRNP D40 on HPV16 mRNA splicing was monitored by HPV16 RT-PCR as described in (B). (**K**) Interactions between hnRNP D40 and cellular spliceosome factors U1-70K, U2AF65 and U2AF35 were investigated by co-immunoprecipitation (Co-IP) assay. Indicated FLAG-tagged, wild-type or mutant hnRNP D40 expression plasmid was transfected into HeLa cells. Whole cell extracts were subjected to IP by anti-FLAG antibody to purify the FLAG-protein interactome complex, followed by western blotting to detect the presence of endogenous cellular spliceosome factors in the complex. Band intensity was quantitated and IP efficiency was calculated as IP band intensity over corresponding Input band intensity. Fold change over WT D40 of IP efficiency is shown under each western blot image. (**L**) Interactions between hnRNP D40 and HPV16 mRNAs were investigated by UV-crosslinking and immunoprecipitation (CLIP) assay. HPV16 subgenomic plasmid pC97ELsLuc was cotransfected with FLAG-tagged wild-type or mutant hnRNP D40 expression plasmid into HeLa cells. UV-crosslinked whole cell extracts were subjected to IP by anti-FLAG antibody followed by extraction of RNA from the FLAG-protein interactome complex and RT-PCR using HPV16-specific RT-PCR primers, 97S+438AS or TotalE6F+TotalE6R. The schematic depiction of the location of the HPV16-specific RT-PCR primers is shown below the gel image. Fold change of CLIP efficiency of wild-type and mutant hnRNP D40s over wild-type D40 was calculated as described in (K).

In an effort to elucidate how hnRNP D40 inhibits HPV16 mRNA splicing, we investigated the effect of deletion mutations in hnRNP D40 (Figure 4A). Analysis of FLAG-tagged, wild-type and mutant hnRNP D40-proteins revealed that all mutants were expressed in the transfected HeLa cells (Figure 4B). To determine the subcellular localization of these hnRNP D40 proteins, a set of plasmids in which the hnRNP D40 open reading frame was fused to EGFP was also created. All hnRNP D40 proteins were localized to the nucleus, although mutant D8 differed from other mutants by showing stronger cytoplasmic than nuclear localization (Figure 4C and Supplementary Figure S3A). Wild-type hnRNP D40 and the N-terminally deleted proteins (D1, D2 and D5) were present primarily in the nucleus, whereas C-terminally deleted proteins (D3, D4, D7 and D9) also showed substantial cytoplasmic staining (Figure 4C and Supplementary Figure S3A). The increased cytoplasmic localization of the C-terminally deleted proteins was a result of deletion of the RGG-domain as substitutions of the arginines in four RGG-motifs for alanine had the same effect (Figure 5A and Supplementary Figure S4B). We concluded that the nuclear localization of the mutant hnRNP D40 proteins allowed us to determine the effect of these proteins on HPV16 mRNA splicing.

Co-transfection of HPV16 reporter plasmid pC97ELsLuc with hnRNP D40 or either of the N-terminally deleted hnRNP D40 plasmids (D1, D2, D5 and D8) (Figure 4D) revealed that all deletions reduced the ability of hnRNP D40 to promote production of HPV16 intron-retained E6 mRNAs (Figure 4D,H) and intron-retained E1 mRNAs (Figure 4E,I). Quantitated percent of all isoforms is shown in Supplementary Figure S3B and C. Longer exposed images of Figure 4E,G are available in Supplementary Figure S3D. Quantitation showed that the reduction in splicing was statistically significant (Figure 4H,I). More specifically, we found that deletion of the N-terminal Alanine-rich region of hnRNP D40 (mutant D5) reduced the ability of hnRNP D40 to promote production of intron-retained HPV16 E6 mRNAs (Figure 4D,H). Deletion of the exon-2 coding region, in addition to the alanine-rich region, as in hnRNP D40 mutant D1 further reduced induction of intron-retained E6 mRNA (Figure 4D,H) and intron-retained E1 mRNA (Figure 4E, I) and unexpectedly activated splicing from SD226 to SA526 (226∧526) (Figure 4D and Supplementary Figure S3B). Deletion also of RRM1 domain, as in hnRNP D40 mutant D2, abolished induction of both intron-retained E6 mRNAs (Figure 4D,H) and intron-retained E1 mRNAs (Figure 4E, I) and unexpectedly activated splicing from SD226 to SA409 (226∧409) (Figure 4D and Supplementary Figure S3B). Overexpression of a mutant hnRNP D40 protein consisting only of RRM1 and RRM2 domains (hnRNP D40 mutant D8) had only a minor effect on HPV16 mRNA splicing (Figure 4D,E), which in this case may be explained by the preferential localization of D8 to the cell cytoplasm (Figure 4C). We concluded that the N-terminus of hnRNP D40 contributed significantly to the inhibition of HPV16 early mRNA splicing and production of E1- and E6-mRNAs and that RRM1 domain was of particular importance.

Interestingly, we found that hnRNP D40 mutant D1 unexpectedly gained the ability to activate splicing to HPV16 3′-splice site SA526 (226∧526) (Figure 4D), while retaining some of its ability to inhibit splicing of both E6/E7 mRNAs and E1/E2 mRNAs (Figure 4D,E). These results suggested that deletion of the N-terminal, alanine-rich region and the exon 2 coding sequence weakened the ability of hnRNP D40 to inhibit splicing, while retaining interactions with the splicing machinery, of which the latter was documented by the enhanced splicing to SA526. Hence, interactions of hnRNP D40 with the splicing machinery and its ability to inhibit splicing could be separated. This interpretation was reinforced by the phenotype of mutant D2 that lost much of its inhibitory effect on the E6/E7 mRNA splicing (Figure 4D,H) and all of its inhibitory effect on E1 mRNA splicing (Figure 4E,I) but gained the ability to promote splicing to HPV16 SA409 (226∧409) (Figure 4D). To determine if similar results were obtained in cells other than HeLa cells, we co-transfected pC97ELsLuc plasmid into 293T cells in the presence of hnRNP D40 or the two N-terminal deletions of hnRNP D40 D1 and D2. Similar results were obtained in the two cell lines (Supplementary Figure S4A): D1 and D2 had lost much of their splicing inhibitory function and activated alternative splicing of the HPV16 E6/E7 mRNAs (Supplementary Figure S4A). RRM1 and RRM2 apparently played a decisive role in the selection of HPV16 splice site since the presence of RRM1 and RRM2 in D1 activated splicing to HPV16 SA526 (226∧526) (Figure 4D), whereas RRM2 alone in D2 activated splicing to HPV16 SA409 (226∧409) (Figure 4D). We concluded that hnRNP D40 RRM1 was essential for efficient splicing inhibition and induction of intron-retained HPV16 E1 and E6 mRNAs and that the N-terminal alanine-rich region and the exon-2 region of hnRNP D40 contributed to splicing inhibition possibly via different mechanisms.

### Nested C-terminal deletions in hnRNP D gradually reduced ability of hnRNP D to inhibit HPV16 mRNA splicing

In contrast to N-terminal deletions of hnRNP D40, either of the C-terminally deleted hnRNP D40 plasmids D3, D4, D7 and D9 (Figure 4A) promoted production of intron-retained E6 mRNAs (Figure 4F, J), although less efficiently than wild-type hnRNP D40. Inhibition of splicing to SA409 and in particular to SA742 was gradually reduced with larger C-terminal deletions in hnRNP D40 (Figure 4F and Supplementary Figure S3B). Even plasmid D9 that contained only the N-terminal alanine-rich region and the exon 2 coding region retained some splicing inhibitory activity (Figure 4F,J). Similarly, splicing inhibition of the HPV16 E1/E2 mRNAs was gradually lost with increasing size of hnRNP D40 C-terminal deletions (Figure 4G,K). Similar results were obtained with the two-C-terminal deletions D3 and D4 in other cell line (Supplementary Figure S4A), corroborating the observation that C-terminal deletions reduced the inhibitory effect on alternative splicing of HPV16 E6/E7 mRNAs. Taken together, these results supported the idea that the N-terminal region of hnRNP D40 played an important role in inhibition of HPV16 E1/E2 and E6/E7 mRNA splicing and indicated that this effect was enhanced by the C-terminus, suggesting that the C-terminus of hnRNP D40 may interact with the splicing machinery.

### The RGG-domain of hnRNP D40 contributes to its ability to inhibit HPV16 mRNA splicing

In an effort to understand how hnRNP D40 interacted with the splicing machinery, we utilized hnRNP D40 mutants D1 and D2 for further mutagenesis. D1 and D2 have N-terminal deletions but retain either both RRM1 and RRM2 (D1), or only RRM2 (D2). Both D1 and D2 contain an intact C-terminus with the Q-rich region and the RG/RGG-rich ‘RGG’-region (Figure 5A). We separately introduced point mutations in the glutamine-rich region (Q) and in the RGG-region in D1 and D2 (Figure 5A). Substitutions of arginine for alanine in four RG/RGG motifs in the RGG-region in D1 (D1-AGG) did not affect the ability of D1 to promote production of intron-retained E6 mRNA (Figure 5B,D) but abrogated the splicing enhancing effect to HPV16 SA526 (226∧526) (Figure 5B,F) and reduced the ability of D1 to promote production of intron-retained E1 mRNAs (Figure 5C, E). In contrast, substitutions of all six glutamines in the Q-rich region for alanine (D1-Q6A), did not affect the ability of D1 to enhance splicing to HPV16 SA526 (Figure 5B,F), nor did they affect the ability of D1 to promote production of intron-retained E1 mRNAs (Figure 5C,E). We concluded that the hnRNP D40 RGG-region was important for enhancement of E6 mRNA splicing to SA526, as well as for inhibition of E1 mRNA splicing. These results were supported by the analysis of hnRNP D40-D2 with the same mutations. Mutations in the RGG domain in D2 (D2-AGG) (Figure 5A) alleviated splicing enhancement of SA409 (Figure 5B,G). Interestingly, both D1-AGG and D2-AGG restored HPV16 E6 mRNA splicing to HPV16 SA742 (226∧742) compared to their corresponding parental mutants, D1 and D2, respectively (Figure 5B,H), which is in line with the observation in Figure 4F that sequential C-terminal deletions restored 226∧742. The D2-AGG mutations did not substantially affect production of intron-retained E1 mRNAs (Figure 5C,E) or intron-retained E6 mRNAs (Figure 5B,D). Similarly to D1-Q6A and D1, D2-Q6A and D2 had similar phenotype (Figure 5F, G, H). Our results demonstrated that the RGG-domain contributed to the ability of D1 and D2 to activate splicing to alternative HPV16 splice sites in the absence of the splicing inhibitory part in the N-terminus of full-length hnRNP D40. We speculated that the C-terminus of hnRNP D40 interacted with the splicing machinery and that the inhibition of splicing and the interactions with the splicing machinery were two separate properties of hnRNP D40. Finally, substitutions of arginine for alanine in the RGG-region in the context of full-length hnRNP D40 showed a reduction of the splicing inhibitory effect of hnRNP D40 on E6 and E1 mRNAs (Figure 5I,J). The effects in the context of the full-length protein were relatively subtle but reproducible and significant (Supplementary Figure S4C). Taken together, our results suggested that the C-terminal RGG-domain of hnRNP D40 contributed to the splicing inhibitory function of hnRNP D40 by interacting with the splicing machinery, while the inhibitory effect on splicing was mediated by the N-terminus of hnRNP D40.

### hnRNP D40 interacts with complex A components of the splicing machinery

To determine if hnRNP D40 interacted with the splicing machinery, we investigated if hnRNP D40 co-immunoprecipitated components of the cellular spliceosome. HeLa cells were transfected with plasmids expressing full-length (D40) or D1 or D2 mutants of hnRNP D40. Cell extracts were prepared and subjected to immunoprecipitation by anti-FLAG antibody followed by western blotting with antibodies to spliceosome components U1-70K, U2AF65 or U2AF35, or with anti-FLAG antibody. Our results revealed that wild-type hnRNP D40 co-immunoprecipitated cellular U1 snRNP component U1-70K and U2 auxiliary factors U2AF65 and U2AF35 (Figure 5K). The two N-terminal mutants of hnRNP D40, D1 and D2, retained the interactions with cellular U1 snRNP components U1-70K and U2AF65 and U2AF35, albeit less efficiently than full-length hnRNP D40 (Figure 5K). Furthermore, the branch-point adenosine recognizing complex component, splicing factor 3b (SF3b) and one of the translation regulators, cytosolic poly(A)-binding protein-1 (PABP-C1) interacted with full-length hnRNP D40 as well as with the N-terminal mutants D1 and D2 (Supplementary Figure S4D). These results not only demonstrated that the C-terminus of hnRNP D40 was sufficient for interactions with these spliceosomal complex A components as well as with downstream RNA-processing regulators but also revealed that the N- terminus of hnRNP D40 contributed to the efficiency of these interactions. We concluded that hnRNP D40 protein interacted with complex A components of the splicing machinery.

### hnRNP D40 interacts with HPV16 mRNAs in an hnRNP D40 RRM1-dependent manner

Next, we investigated if hnRNP D40 or deletion mutants thereof (D1 and D2) interacted with HPV16 RNA. Plasmids expressing FLAG-tagged hnRNP D40, D1 or D2 were co-transfected with HPV16 reporter plasmid pC97ELsLuc into HeLa cells followed by UV-crosslinking and Immunoprecipitation (CLIP) with affinity purification of FLAG tagged hnRNP D40-RNA complexes. FLAG-antibody-immunoprecipitated UV-crosslinked RNA was extracted and subjected to RT-PCR with HPV16 specific primers (Figure 5L). The results revealed that immunoprecipitation of wild-type hnRNP D40 also immunoprecipitated HPV16 RNA detected by HPV16 primer pair TotalE6F+TotalE6R that amplifies cDNA representing all HPV16 mRNAs transcribed from pC97ELsLuc (Figure 5L). Analysis of the HPV16 RNA with primer pair 97S+438AS that discriminates between intron-retained and spliced HPV16 E6 mRNAs revealed that hnRNP D40 interacted with both intron-retained and spliced E6 mRNAs (Figure 5L). Deletion of N-terminal alanine-rich and exon-2 region (D1 mutant) did not affect the interactions with HPV16 RNA but deletion of RRM1 did (D2 mutant) (Figure 5L). We concluded that hnRNP D40 interacts with HPV16 mRNAs directly in an hnRNP D40 RRM1-dependent manner.

### hnRNP D40 interacts with the entire intronic region of the E6 coding region as well as with a previously identified HPV16 splicing silencer in E7 region

**Figure 6.**
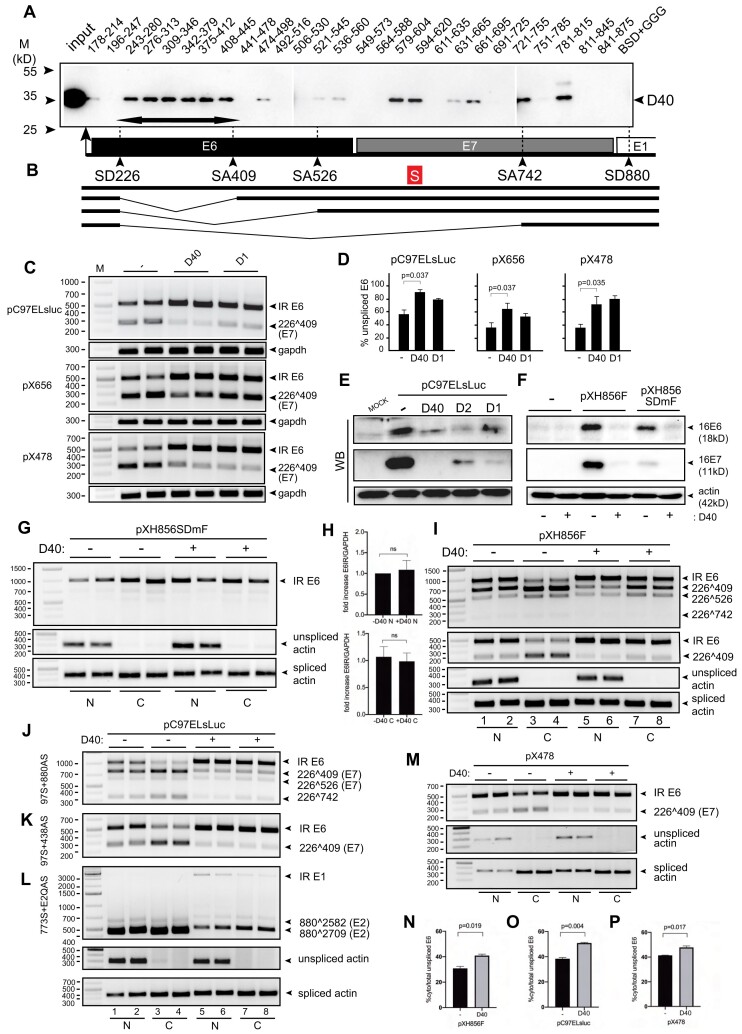
hnRNP D40 interacted with HPV16 E6E7 RNA sequences *in vitro* and influenced HPV16 E6E7 mRNA processing. (**A**) RNA oligonucleotide-mediated pull-down assay using sequential short RNA oligos spanning the entire HPV16 E6- and E7- coding regions. The numbers in each RNA-oligo name represent HPV16 nucleotide positions and refer to nucleotide numbers in the reference HPV16 genome (GenBank: K02718.1). Thick double-headed arrow: intronic region of E6/E7 mRNAs located between SD226 and SA409. (**B**) Schematic representation of RNA splicing in the HPV16 E6 and E7 coding region. Intron-retained E6 mRNA is depicted as well as spliced HPV16 mRNAs 226∧409, 226∧526 and 226∧742. Box with S: previously identified E6/E7 mRNA splicing silencer element (46). RNA oligo sequences are available in Supplementary Table S3. (**C**) The effect of hnRNP D40 on the splicing of HPV16 E6E7 mRNA produced from pC97ELsLuc as well as shorter plasmids pX656 and pX478 was monitored by RT-PCR. The schematic maps of pX656 and pX478 plasmids and the positions of RT-PCR primers are shown in Supplementary Figure S5. (**D**) The percentage of intron-retained E6 mRNA over a total sum of all spliced isoform quantitated from (C) are shown. (**E**) HPV16 E6 and E7 proteins expressed in HeLa transfected with pC97ELsLuc together with indicated wild-type or mutant hnRNP D40. Western blotting was performed using anti-HPV16 E6 or E7 specific antibodies. (**F**) HPV16 E6 and E7 proteins produced in 293T cells transfected with pXH856F, which encodes intact HPV16 E6- and E7- coding regions, or with pXH856SDmF, which contains a mutationally inactivated SD226 splice site, in the absence (−) or presence (+) of cotransfected hnRNP D40 expression plasmid. Western blotting was performed with anti-HPV16 E6 or E7 specific antibodies. Wild-type SD226 (GAG|GUAUAUGA: a vertical line indicates 5′ splice site) was changed to (GAG|GCCUAUGA: underline indicates changed nucleotide) in SD226 mutant. Schematic maps of pXH856F and pXH856SDmF are shown in Supplementary Figure S5. (**G**) Subcellular distribution of intron-retained E6 mRNAs produced from pXH856SDmF in the absence (−) or presence (+) of hnRNP D40 plasmid. Nuclear and cytoplasmic fractions were prepared from the transfected cells and RNA was extracted and subjected to HPV16 RT-PCR using primers TotalE6F and 757AS (for primer location, see Figure 1C and Supplementary Figure S5E). Cell fractionation was validated by RT-PCR analysis of unspliced actin RNA located exclusively in the nuclear fraction while spliced actin mRNAs found in both fractions. (**H**) qPCR using cDNA samples from (G) to evaluate levels of intron-retained E6 mRNAs in nuclear and cytoplasmic fractions in the absence or presence of hnRNP D40. Primers TotalE6F and 234AS were used (for primer location, see Figure 1C and Supplementary Figure S5E). (**I**) Nuclear (N) and cytoplasmic (C) distribution of HPV16 alternatively spliced mRNAs produced from pXH856F in the absence (−) or presence (+) of hnRNP D40 plasmid. RT-PCR was performed with primers: 97S+757AS (top panel) or 97S+438AS (second panel). Primer locations are shown in Figure 1C and Supplementary Figure S5D. (**J–L**) Nuclear (N) and cytoplasmic (C) distribution of HPV16 alternatively spliced mRNAs produced from pC97ELsLuc in the absence (−) or presence (+) of cotransfected hnRNP D40 plasmid. RT-PCR was performed with the following primers: (J) 97S+880AS, (K) 97S+438AS or (**L**) 773S+E2QAS. (**M**) Nuclear (N) or cytoplasmic (C) distribution of HPV16 alternatively spliced mRNAs produced from HPV16 plasmid pX478 in the absence (−) or presence (+) of cotransfected hnRNP D40 plasmid, followed by RT-PCR using primers, 97S+438AS. Schematic map of plasmid pX478 and location of HPV16 RT-PCR primers are shown in Supplementary Figure S5C. (**N–P**) Percentage of cytoplasmic intron-retained E6 mRNAs over the total sum of nuclear and cytoplasmic intron-retained E6 mRNAs in the absence (−) or presence of hnRNP D40 quantitated on (N) pXH856F from (I), (O) pC97ELsLuc from (K) or (P) pX478 from (M). Student t-test was executed and obtained *P* values were displayed. n.s., no significance.

Having established that hnRNP D40 interacts with HPV16 mRNAs (Figure 5L) and with components of the splicing machinery (Figure 5K) thereby inhibiting HPV16 early mRNA splicing and promoting intron retention and production of intron-retained HPV16 E1 and E6 mRNAs, we wished to identify the HPV16 sequences that were targeted by hnRNP D40. For this purpose, we focused on the E6/E7 coding region as it is shorter than the E1 coding region. To this end, we performed RNA-mediated pull downs of hnRNP D40 with overlapping, biotinylated RNA oligonucleotides that covered the E6- and E7-coding regions from HPV16 nucleotide position 178 to 875 (Figure 6A,B). This experimental approach was chosen since hnRNP D40 may not necessarily bind directly to HPV16 mRNAs. Cell extracts were prepared from HeLa cells transfected with FLAG-tagged hnRNP D40. These extracts were subjected to RNA-mediated protein pull downs with the RNA oligos indicated in Figure 6A,B, followed by western blotting with anti-FLAG antibody (Figure 6A,B). The results revealed that hnRNP D40 was specifically pulled down by a subset of the RNA oligos. Of particular interest was that all RNA oligos spanning the E6-encoding intron between HPV16 5′-splice site SD226 and 3′-splice site SA409 pulled down hnRNP D40 (Figure 6A,B, highlighted with a double-headed arrow). Furthermore, RNA oligos 579-604 and 594-620 that spanned a previously identified splicing silencer (46) also pulled down hnRNP D40 (Figure 6A,B). Additional RNA oligos that pulled down hnRNP D40 did not coincide with known RNA elements except for oligos 721-755 that encoded HPV16 3′-splice site SA742 (Figure 6A,B). We concluded that interactions of hnRNP D40 with a previously identified splicing silencer located between HPV16 nucleotide positions 594 and 604, as well as with the intronic sequences in the E6 coding region (243-445), correlated with the ability of hnRNP D40 to inhibit splicing between SD226 and SA409 and to promote retention of the intron between the same two splice sites.

### hnRNP D40 promotes intron retention of HPV16 E6 mRNAs independently of the HPV16 E1 sequences

Since hnRNP D40 interacted with sequences in the E6 coding region, we investigated if hnRNP D40 could inhibit E6 mRNA splicing and induce intron retention of the E6 mRNAs in the absence of E1 sequences. We had previously showed that hnRNP D40 inhibited splicing and induced intron retention of the E1/E2 mRNAs in the absence of E6 and E7 sequences (see effect of hnRNP D40 on pBELsLuc (Figure 3E–J, Supplementary Figure S2)). To this end we co-transfected hnRNP D40 plasmid with HPV16 plasmid pX656 that starts at HPV16 nucleotide position 97 and ends at HPV16 position 656 and does not overlap with the previously used HPV16 plasmid pBELsLuc (Supplementary Figure S5A and B). hnRNP D40 inhibited splicing of HPV16 E6/E7 mRNAs produced from pX656, albeit less efficiently than it inhibited splicing of E6/E7 mRNAs produced from pC97ELsLuc (Figure 6C,D), demonstrating that hnRNP D40 can inhibit splicing in the HPV16 E6/E7-coding region independently of E1 sequences. The pX656 contained a previously identified splicing silencer located between HPV16 positions 594 and 604 in addition to the E6-encoding intron with HPV16 splice sites SD226, SA409 and SA526, but the pull-down experiments displayed in Figure 6A demonstrated that the major sites of interactions between hnRNP D40 and HPV16 E6/E7 mRNAs were within the E6 intron between SD226 and SA409. Thus, our results predicted that a smaller plasmid containing only a part of the E6 coding region encompassing SD226, SA409 and the E6 intron (without splicing silencer in the E7 coding region) should also respond to hnRNP D40 overexpression. HPV16 plasmid pX478 that contains HPV16 sequences from HPV16 nucleotide position 97 to position 478 (Supplementary Figure S5C) was transfected into HeLa cells in the absence or presence of hnRNP D40 expression plasmid. RT-PCR analysis of HPV16 mRNAs revealed that hnRNP D40 inhibited splicing and promoted production of intron-retained HPV16 mRNAs from this minimal HPV16 pX478 expression plasmid (Figure 6C,D). As expected, hnRNP D40 deletion mutant D1 also inhibited splicing of the HPV16 mRNAs produced from plasmid pX478 but less efficiently than wild-type hnRNP D40 (Figure 6C,D). We concluded that hnRNP D40 promoted intron retention of HPV16 E6 mRNAs independently of HPV16 E1 sequences.

### hnRNP D40 increases the levels of HPV16 intron-retained E6 mRNAs in the cytoplasm

Our results also suggested that hnRNP D40 should increase E6 protein production at the expense of E7 protein production as a result of its splicing inhibitory function of HPV16 E6/E7 mRNAs. Analysis of HPV16 E6 and E7 protein production from pC97ELsLuc transfected in the absence or presence of hnRNP D40 expression plasmid unexpectedly revealed that overexpression of hnRNP D40 suppressed production of both E6 and E7 protein (Figure 6E). Similar results were obtained with hnRNP D40 mutant D1 (Figure 6E). hnRNP D40 also inhibited E6 and E7 protein production from a smaller HPV16 expression plasmid named pXH856F that encodes only E6 and E7 (Figure 6F and Supplementary Figure S5D). This effect was independent of the splicing process itself since overexpression of hnRNP D40 inhibited E6 and E7 protein production also from a version of the pXH856F plasmid in which the major HPV16 5′splice site SD226 was mutationally inactivated, plasmid pXH856SDmF (Figure 6F and Supplementary Figure S5E). These results suggested that hnRNP D40 was associated with HPV16 mRNAs also in the absence of RNA splicing and as such, could potentially affect other RNA processing steps, thereby explaining the reduction in E6 protein levels despite the increase in the levels of intron-retained E6 mRNAs.

To determine if hnRNP D40 affected export of intron-retained E6 mRNAs, we analyzed the subcellular distribution of the intron-retained E6 mRNAs produced from the splicing deficient plasmid pXH856SDmF in the absence or presence of hnRNP D40. The intron-retained E6 mRNAs produced from pXH856SDmF were present in the nucleus and in the cytoplasm (Figure 6G). Unexpectedly, the levels and subcellular distribution of E6 mRNAs were similar in the absence or presence of hnRNP D40 (Figure 6G,H). RT-PCR of intron-retained and spliced actin RNA served as a control for proper fractionation of the transfected cells (Figure 6G). We concluded that the nuclear export of intron-retained E6 mRNAs transcribed from the splicing deficient plasmid pXH856SDmF was not considerably affected by hnRNP D40 (Figure 6G,H). Thus, inefficient nuclear mRNA export of intron-retained E6 mRNA in the presence of hnRNP D40 could not explain the significant inhibition of E6 protein production exerted by hnRNP D40 (Figure 6F). To exclude the possibility that the mutation of HPV16 5′-splice site SD226 in pXH856SDmF affected the RNA export regulation mediated by hnRNP D40, the effect of hnRNP D40 on the subcellular distribution of E6 and E7 mRNAs was investigated using pXH856F encoding wild-type SD226. The inhibitory effect of hnRNP D40 on HPV16 E6/E7 mRNA splicing was observed however the effect appeared to be mainly against 226∧409 (Figure 6I). We observed that the intron-retained E6 mRNAs were to a large extent restricted to the nuclear fraction while the levels of the spliced, HPV16 226∧409-mRNAs were higher in the cytoplasm than in the nucleus (Figure 6I, top panel, lanes 1–4). In the presence of hnRNP D40, primarily intron-retained E6 mRNAs were detected both in the nucleus and cytoplasm (Figure 6I, top panel, lanes 5–8). The results were confirmed using a primer pair that only detected intron-retained E6 mRNAs and mRNAs spliced between splice sites SD226 and SA409 (226∧409) (Figure 6I, second panel), demonstrating a statistically significant increase of cytoplasmic intron-retained E6 distribution in the presence of hnRNP D40 (Figure 6N). These results indicated that hnRNP D40 did not inhibit the nuclear export of intron-retained E6 mRNAs. Thus, inhibition of nuclear export of intron-retained E6 mRNAs could not explain the reduction of E6 protein levels in the presence of hnRNP D40 observed in Figure 6E and F. This observation was consistent with results obtained using HPV16 subgenomic plasmid pC97ELsLuc (Figure 6J, K and O). Furthermore, we showed that hnRNP D40 enhanced the levels of cytoplasmic intron-retained E6 mRNAs also from the smaller HPV16 subgenomic plasmid pX478 (Figure 6M). This increase was statistically significant (Figure 6P). Taken together, we concluded hnRNP D40 had multiple impacts and affected various steps of HPV16 E6 and E7 mRNA processing: hnRNP D40 interacted with E6/E7 mRNAs at multiple positions, it inhibited E6/E7 mRNA splicing independently of the E1 region, and increased levels of intron-retained E6 mRNAs in the cytoplasm. However, hnRNP D40 also significantly reduced the levels of E6 protein produced from the intron-retained E6 mRNAs.

### hnRNP D40 increased the levels of HPV16 intron-retained E1 mRNAs in the cytoplasm

**Figure 7.**
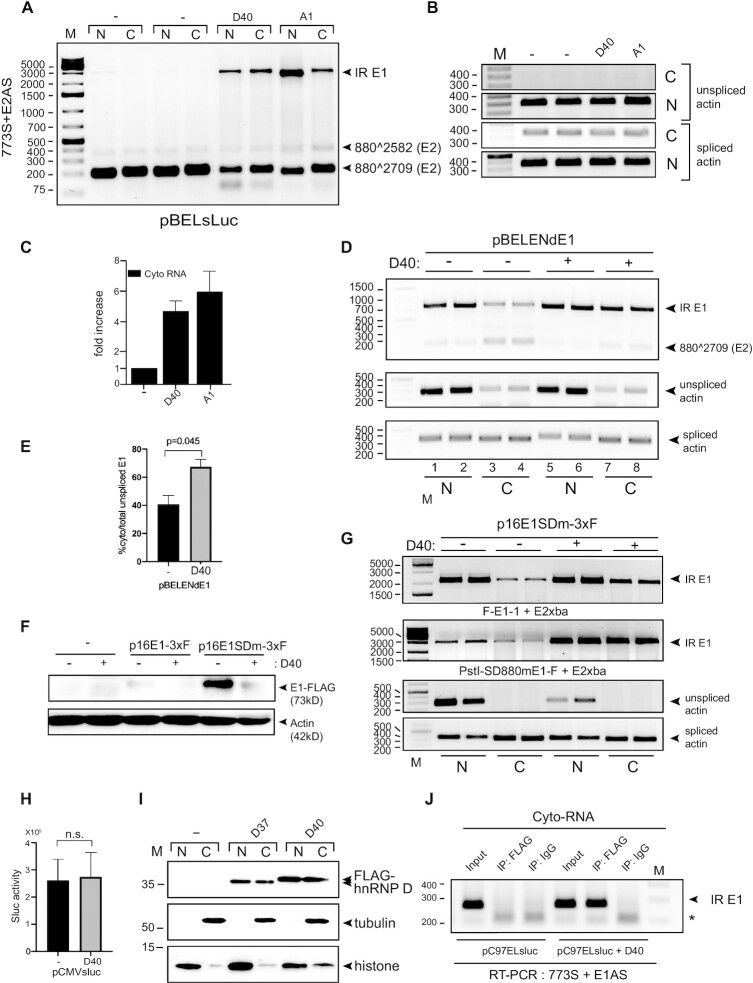
hnRNP D40 upregulated levels of HPV16 intron-retained E1 mRNAs in the cytoplasm and interacts with these mRNAs in the cytoplasmic fraction. (**A**) HeLa cells were transfected with pBELsluc plasmid in the absence (−) or presence of plasmids expressing either hnRNP D40 or hnRNP A1. Nuclear (N) and cytoplasmic (C) fractions were prepared from the transfected cells and RNA was extracted and subjected to HPV16-specific RT-PCR using primer pair 773S+E2AS. (**B**) Cellular fractionation was validated by analysis by RT-PCR of unspliced and spliced actin mRNAs. (**C**) Levels of HPV16 intron-retained E1 mRNAs in the cytoplasmic fraction was determined by RT-PCR with primers 773S+E1AS. Quantification of electrophoresed RT-PCR products were performed as described and normalized to RT-PCR products representing cytoplasmic spliced actin mRNA. Fold over control (−) is shown. (**D**) Effect hnRNP D40 on subcellular localization of HPV16 intron-retained E1 mRNAs and spliced E2 mRNAs produced from HPV16 subgenomic plasmid pBELENdE1 (for schematic representation of pBELENdE1, see Supplementary Figure S7C). pBELENdE1 was transfected into HeLa cells in the absence (−) or presence (+) of hnRNP D40 plasmid. RT-PCR was performed on nuclear or cytoplasmic RNA using HPV16-specific primers pair 773S+E2Xba that detects HPV16 intron-retained E1 mRNAs as well as spliced mRNAs (880∧2709). (**E**) Percentage cytoplasmic intron-retained HPV16 E1 mRNAs over a total sum of nuclear and cytoplasmic intron-retained E1 mRNAs in absence (−) or presence of hnRNP D40 from (D). (**F**) Effect of hnRNP D40 on HPV16 E1 protein levels was determined by western blotting on extracts form HeLa cells transfected with HPV16 E1-FLAG expressing plasmid p16E1-3xF (wild type) or p16E1SDm-3xF (harboring mutations at splice donors SD880 and SD1302 in the E1 gene thereby abolishing E2 mRNA splicing), in the absence (−) or presence (+) of hnRNP D40 expressing plasmid. Western blotting using anti-FLAG antibody (M2). (**G**) The levels of HPV16 intron-retained E1 mRNAs produced by p16E1SDm-3xF in nuclear and cytoplasmic fractions, in the absence (−) or presence (+) of hnRNP D40 expression plasmid, was determined by HPV16-specific RT-PCR primers primer pairs F-E1-1+E2Xba and PstI-SD880mE1-F+E2Xba (primer positions are displayed in Supplementary Figure S7). (**H**) Overexpression of hnRNP D40 does not affect production of sLuc from a CMV-promoter driven sLuc gene. (**I**) HeLa cells were transfected with indicated, FLAG-tagged hnRNP D expression plasmids. Nuclear and cytoplasmic extracts were subjected to Western blotting with anti-FLAG antibody or anti-tubulin or anti-histone antibody to control for subcellular fractionation. (**J**) Association between HPV16 E1 mRNAs and hnRNP D40 protein in the cytoplasm demonstrated by CLIP assay on cytoplasmic extracts from HeLa cells transfected with HPV16 subgenomic plasmid pC97ELsLuc in the absence or presence of FLAG-hnRNP D40 expressing plasmid. Cytoplasmic extracts were subjected to immunoprecipitation with anti-FLAG antibody to purify FLAG-hnRNP D40: RNA complexes. RNA in the ribonucleoprotein (RNP) complex was extracted and subjected to RT-PCR using primers 773S and E1AS that specifically detect HPV16 intron-retained E1 mRNAs.

In addition to the intron-retained E6 mRNAs produced from pC97ELsLuc, intron-retained E1 mRNAs transcribed from pC97ELsLuc were also increased in both nucleus and cytoplasm (Figure 6L). To confirm this effect also in the absence of E6/E7 sequences on the mRNAs, we used HPV16 subgenomic reporter plasmid pBELsLuc that lacks E6E7 genes (Figure 3E). pBELsLuc was transfected into HeLa cells in the absence or presence of hnRNP D40 plasmid followed by fractionation into nuclear and cytoplasmic fractions prior to RNA extraction and RT-PCR. As can be seen in Figure 7A, spliced E2 mRNA (880∧2709) levels were reduced in the presence of hnRNP D40, as expected, but they were present in both nuclear and cytoplasmic fractions, although predominantly in the cytoplasm. In sharp contrast to the spliced E2 mRNAs, the intron-retained E1 mRNAs were detected primarily in the presence of hnRNP D40 (Figure 7A). In the presence of hnRNP D40, the intron-retained E1 mRNAs were present in both the nuclear and cytoplasmic fractions (Figure 7A), demonstrating that hnRNP D40 not only inhibited splicing of the HPV16 E1/E2 mRNAs and promoted intron retention to generate intron-retained E1 mRNAs, it also allowed these mRNAs to be exported to the cytoplasm. A similar effect was observed with hnRNP A1 (Figure 7A) an hnRNP protein evolutionarily close to hnRNP D protein family (54) and initially shown to inhibit HPV16 E6/E7 and E1/E2 mRNA splicing (Figure 2A,C). Actin mRNAs served as controls for cellular fractionation: unspliced actin mRNAs were present primarily in the nuclear fraction, whereas spliced actin mRNAs were detected in both fractions (Figure 7B). Nuclear restricted Lamin B and SRSF2 and the shuttling SRSF1 protein served as additional controls for cellular fractionation (Supplementary Figure S6). We also performed RT-qPCR with primers 773s and E1AS on the cytoplasmic fractions, a primer pair that is specific for intron-retained HPV16 E1 mRNAs (Figure 1C). The levels of HPV16 intron-retained E1 mRNAs in the cytoplasm increased more than four-fold in the presence of hnRNP D40 (Figure 7C). We concluded that hnRNP D40 inhibited HPV16 E1/E2 mRNA splicing, promoted intron retention to generate intron-retained E1 mRNAs and enhanced the appearance of the E1 mRNAs in the cytoplasm independently of E6/E7 sequences on the mRNAs.

The HPV16 subgenomic plasmid pBELEN encodes only the HPV 16 E1 gene including all splice sites in the E1 coding region (Supplementary Figure S7B). pBELEN responded to hnRNP D40 by enhanced production of intron-retained E1 mRNA (Supplementary Figure S7D). RT-PCR analysis of HPV16 mRNAs produced from pBELEN in the absence or presence of hnRNP D40, revealed that the level of HPV16 intron-retained E1 mRNA increased in both nucleus and cytoplasm, whereas the levels of HPV16 spliced E2 mRNAs (880∧2709) were reduced in the presence of hnRNP D40 (Supplementary Figure S7E). This result indicated that redistribution of intron-retained E1 mRNA to the cytoplasm was enhanced in the presence of hnRNP D40 (Supplementary Figure S7F). For further confirmation, we generated a plasmid that produced a shorter intron-retained E1 mRNA that would be easier to detect and quantitate by RT-PCR since the predicted size of the entire intron-retained E1 mRNA produced from pBELEN is relatively big (>2 kb). We therefore introduced a deletion in the E1 coding region in pBELEN, between the HPV16 major splice sites SD880 and SA2709, to generate a smaller plasmid named pBELENdE1 (Supplementary Figure S7C). Detection of intron-retained ‘E1’ mRNAs was considerably improved when pBELENdE1 was used (Figure 7D lanes 1–4, compared to Figure 7A). RT-PCR analysis of HPV16 mRNAs produced from pBELENdE1 in the absence or presence of hnRNP D40, revealed that the levels of HPV16 intron-retained mRNAs were increased in both nucleus and cytoplasm (Figure 7D), an increase that was statistically significant (Figure 7E), whereas the levels of HPV16 spliced E2 mRNAs (880∧2709) were reduced in the presence of hnRNP D40, and were located primarily in the cytoplasmic fraction (Figure 7D). Of particular interest was that the proportion of cytoplasmic intron-retained HPV16 mRNAs was significantly increased in the presence of hnRNP D40 (Figure 7E). These results confirmed that hnRNP D40 inhibited HPV16 mRNA splicing and promoted intron retention to generate intron-retained HPV16 E1 mRNAs that were exported to the cytoplasm.

Finally, we wished to determine if the increase of intron-retained E1 mRNAs in the cytoplasm caused by hnRNP D40 also resulted in increased E1 protein levels. We generated an HPV16 E1 expression plasmid in which the E1 gene was fused with three-times FLAG sequence at its 3′-end named p16E1-3xF (Supplementary Figure S7G). This plasmid contained intact HPV16 5′-splice sites SD880 and SD1302 and had the potential to produce an intron-retained mRNA encoding a 73kD, 3xFLAG-tagged HPV16 E1 protein, as well as mRNAs spliced to HPV16 3′-splice site SA2709. In a second version of this plasmid, SD880 and SD1302 were mutationally inactivated to generate plasmid p16E1SDm-3xF (Supplementary Figure S7H). As expected, HPV16 E1 protein production was greatly improved from p16E1SDm-3xF compared to p16E1-3xF (Figure 7F), but overexpression of hnRNP D40 decreased E1 protein levels from both plasmid p16E1-3xF and p16E1SDm-3xF (Figure 7F). Analysis of the mRNAs produced by p16E1-3xF and p16E1SDm-3xF revealed that intron-retained HPV16 E1 mRNA levels transcribed from p16E1SDm-3xF were increased in the cytoplasm in the presence of hnRNP D40 (Figure 7G). The results were confirmed by using different sets of primer pairs (the primer positions are indicated in Supplementary Figure S7H). The results revealed that the levels of intron-retained E1 mRNAs increased in the presence of hnRNP D40 even though HPV16 5′-splice sites SD880 and SD1302 had been inactivated (Figure 7G), which indicated that hnRNP D40 also functioned independently of splicing. This effect was not due to transcriptional activation by hnRNP D40 since hnRNP D40 does not affect transcription from CMV-promoter driven plasmids (Figure 7H). Taken together, these results were reminiscent of the effect of hnRNP D40 on the HPV16 E6 mRNA and E6 protein levels (Figure 6). Our results indicated that hnRNP D40, in addition to its splicing inhibitory function, interacted with HPV16 E1 mRNAs also in the absence of RNA splicing and promoted the appearance of intron-retained HPV16 E1 mRNAs in the cytoplasm. However, these mRNAs were poorly translated into E1 protein and the results suggested that hnRNP D40 negatively interfered with mRNA translation. Indeed, translation *in vitro* of both E6 and E1 mRNAs into E6 and E1 proteins was inhibited by recombinant hnRNP D protein but not by bovine serum albumin (BSA) (Supplementary Figure S8A, B, D and E). This translational inhibition was specific for HPV16 E6 and E1 mRNAs since luciferase mRNA translation was unaffected by the presence of recombinant hnRNP D protein (Supplementary Figure S8C).

### hnRNP D40 is associated with HPV16 mRNAs in the cytoplasm

The results presented above suggested that hnRNP D not only inhibited HPV16 mRNA splicing but also might have the ability to accompany the HPV16 mRNAs to the cytoplasm. As already shown in Figure 4C, overexpressed hnRNP D40-EGFP was primarily located in the nucleus. However, hnRNP D is known to be a shuttling protein and small amounts of hnRNP D40 could potentially be present in the cytoplasm. We therefore transfected HeLa cells with a plasmid expressing a FLAG-tagged hnRNP D40 protein, fractionated the cells and analyzed nuclear and cytoplasmic fractions for FLAG-tagged hnRNP D40 by western blotting. The results revealed that the FLAG-tagged hnRNP D40 produced from the expression plasmid could be detected in both nuclear and cytoplasmic compartments (Figure 7I), whereas tubulin was detected primarily in the cytoplasmic- and histone in the nuclear-fractions (Figure 7I). To determine if hnRNP D40 was associated with the intron-retained HPV16 E1 mRNAs in the cytoplasm, we performed a CLIP assay on cytoplasmic extracts from HeLa cells transfected with HPV16 reporter plasmid pC97ELsLuc in the absence or presence of transfected pFLAG-hnRNP D40 plasmid. As can be seen from the results, intron-retained HPV16 mRNAs could be specifically amplified from the UV-crosslinked RNA–protein complexes immunoprecipitated with anti-FLAG antibody (Figure 7J). These results demonstrated that HPV16 intron-retained E1 mRNAs were associated with hnRNP D40 in the cell cytoplasm, suggesting that hnRNP D40 accompanied the intron-retained HPV16 mRNAs from the nucleus to the cytoplasm where it potentially interfered with HPV16 mRNA translation in a negative manner.

### hnRNP D40 promotes intron retention and production of E1 and E6 mRNAs produced from episomal HPV16 genomes in human primary keratinocytes

**Figure 8.**
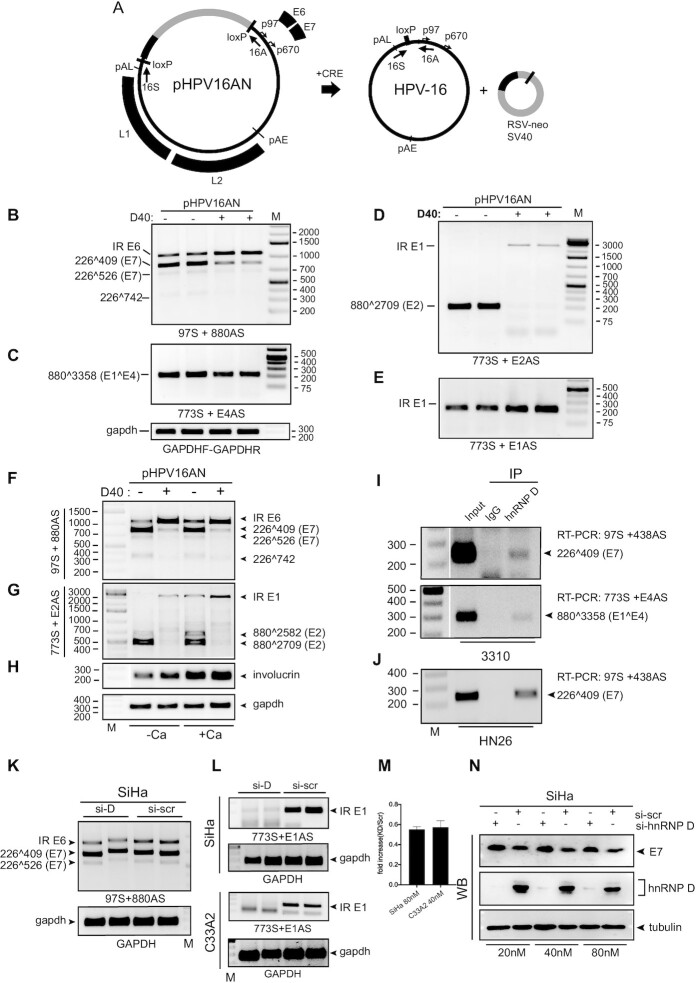
hnRNP D40 inhibits splicing of E6E7 and E1E2 mRNAs transcribed from episomal HPV16 DNA in primary human keratinocytes. (**A**) Schematic representation of the pHPV16AN plasmid and episomal HPV16 DNA production using Cre-loxP transfection system. (**B–E**) RT-PCR on RNA extracted from HeLa cells transfected with pHPV16AN together with Cre recombinase expressing plasmid in the absence (−) or presence (+) of hnRNP D40 expressing plasmid. RNA was extracted and HPV16 RNA splicing was monitored by RT-PCR using primers (B) 97S+880AS, (C) 773S+E4AS, (D) 773S+E2AS and (E) 773S+E1AS. (**F–H**) hnRNP D40 effect on HPV16 mRNA splicing from episomal HPV16 DNA in human primary keratinocytes with or without induction of differentiation. pHPV16AN was transfected into primary normal human foreskin keratinocyte (nHFK) together with Cre recombinase expressing plasmid, in the absence (−) or presence (+) of hnRNP D40 expression plasmid. CaCl_2_ at a final concentration of 2.4 mM was added at 12 h post transfection (+Ca) for 24 h to induce differentiation. RNA was extracted and HPV16 RNA splicing was monitored by RT-PCR using primer pairs indicated to the left of the gel images. (**I** and **J**) Interactions between endogenously expressed HPV16 mRNAs and cellular hnRNP D proteins were analyzed by RIP assay on extracts from HPV16-immortalized human keratinocyte cell line 3310 (56) (**I**) or HPV16-positive tonsillar cancer cell line (55) HN26 cells (**J**). Whole cell extracts were subjected to immunoprecipitation with anti-hnRNP D antibody followed by RNA extraction and RT-PCR with HPV16-specific primers indicated to the right of the gel images. (**K** and **L**) SiHa or C33A2 cells were transfected with siRNAs to hnRNP D (si-hnRNP D) or scrambled siRNA (si-scr). 80 nM siRNA for SiHa cells and 40 nM siRNA for C33A2 cells were transfected. RNA was extracted at 24 h post-transfection to monitor intron-retained E6 mRNAs (K) or at 48 hrs post-transfection to monitor intron-retained E1 mRNAs (L), followed by RT-PCR with indicated primer pairs. (**M**) RT-qPCR to evaluate hnRNP D KD effect on HPV16 intron-retained E1 mRNAs was performed with cDNA from (L). (**N**) WB analysis to evaluate hnRNP D knockdown on HPV16 E7 protein production in SiHa cells transfected with siRNA to hnRNP D (si-hnRNP D) or with scrambled siRNA (si-scr) for 72 h.

Finally, we wished to determine if hnRNP D40 was actively inhibiting splicing also of mRNAs produced from complete, episomal HPV16 genomes. We therefore used plasmid pHPV16AN that encodes the entire HPV16 genome (Figure 8A) (43). The pHPV16AN plasmid was cotransfected with cre-recombinase-expressing plasmid to release the HPV16 genome between the loxP sites and generate the episomal form of the HPV16 genome (Figure 8A). Transfections were performed in the absence or presence of hnRNP D40 expression plasmid. As can be seen in Figure 8B, hnRNP D40 inhibited splicing of the E6/E7 mRNAs and promoted production of intron-retained E6 mRNAs (Figure 8B). A relatively minor inhibitory effect on splicing from SD880 to SA3358 was observed (Figure 8C). Importantly, the inhibitory effect on the E2 mRNAs was readily observed, followed by intron retention and generation of intron-retained E1 mRNAs (Figure 8D). Since transfection of cells with pHPV16AN is relatively inefficient, the larger, intron-retained E1 mRNAs may be relatively inefficiently amplified with primer pair 773S and E2AS. Therefore, we also monitored the levels of intron-retained E1 mRNAs with primers 773S and E1AS (Figure 1E) that also detected an increase in the levels of E1 mRNAs in the presence of hnRNP D40 (Figure 8E). These results were reproduced by transfection of pHPV16AN in the absence or presence of hnRNP D40 plasmid in human primary keratinocytes. As can be seen, hnRNP D40 inhibited splicing and promoted production of intron-retained E6 mRNAs (Figure 8F) and E1 mRNAs (Figure 8G). Notably, induction of calcium-dependent differentiation of the transfected primary keratinocytes increased the levels of intron-retained E1 mRNAs (Figure 8G), an effect that was further enhanced by hnRNP D40 overexpression (Figure 8G). Induction of differentiation of the primary keratinocytes by calcium was confirmed by the increase in the levels of involucrin mRNAs (Figure 8H). Furthermore, we wished to obtain support for the physiological relevance of the interactions between hnRNP D protein and HPV16 mRNAs. To this end we monitored interactions between hnRNP D and HPV16 early mRNAs by RIP assay in the HPV16-positive head-and-neck cancer derived HN26 cells recently established from a tonsillar cancer patient at Lund University hospital (55), and from the in-house HPV16-immortalized keratinocyte cell line 3310 with integrated HPV16 DNA (56). The interactions between hnRNP D and HPV16 early E6/E7 mRNAs were readily detected by RT-PCR in the immunoprecipitated hnRNP D-RNA complexes obtained from either the tonsillar cancer cell line HN26 (Figure 8I) or the HPV16-immortalized human keratinocyte 3310 cell line (Figure 8J). Finally, the effect on HPV16 mRNA splicing and protein expression of small-interfering RNA (siRNA) mediated knockdown (KD) of hnRNP D proteins was determined using HPV16 positive cancer cell line SiHa and the in-house generated cell line C33A2 (42) (Figure 8K–N). Briefly, the C33A2 cell line was derived from C33A cells stably transfected with HPV16 reporter plasmid pBELsLuc plasmid (Figure 3E). KD of hnRNP D revealed a reduction in intron retention on E6 mRNAs in SiHa cells (Figure 8K) as well as a reduction in intron retention on E1 mRNAs both in SiHa and C33A2 cells (Figure 8L,M). Although the effect of hnRNP D knockdown on E6 mRNAs in SiHa cells was weaker than the effect on E1 mRNAs, this decrease was statistically significant (*P* = 0.003). Furthermore, KD of hnRNP D resulted in an increase of HPV16 E7 protein levels in SiHa cells (Figure 8N), which is consistent with an increase in spliced 226∧409 mRNAs that are dedicated for E7 protein production (Figure 8K). Taken together, we concluded that hnRNP D40 promoted production of HPV16 intron-retained E1 and E6 mRNAs with retained introns also on mRNAs expressed from the episomal form of the HPV16 genome suggesting that the interactions of between HPV16 early mRNAs and hnRNP D are of significance also in HPV16 positive cancer cell lines. Furthermore, knockdown of hnRNP D enhanced production of the HPV16 E7 protein in HPV16-driven cervical cancer cells.

## DISCUSSION

Previously, we have shown that HPV16 5′- splice site SD3632 that is exclusively utilized during late stages of the HPV16 life cycle, is suppressed in mitotic cells by binding of hnRNP D proteins to AUAGUA motifs upstream of SD3632 (43). In our current manuscript, we present results that indicate that hnRNP D proteins inhibit HPV16 E1/E2- and E6/E7-mRNA splicing. The effect of hnRNP D on the HPV16 mRNA splicing process (Figure 3) was followed by effects on subsequent steps of HPV16 mRNA processing including a potential increase in mRNA stability of (Figures 6 and 7) and enhanced nuclear export (Figures 6 and 7) of intron-retained HPV16 mRNAs. Consequently, intron-retained HPV16 E1 and E6 mRNA levels in the cytoplasm increased in the presence of hnRNP D. The regulation of RNA processing including alternative pre-mRNA splicing is particularly important for the regulation of HPV16 gene expression since all HPV16 genes are expressed from a plethora of alternatively spliced viral mRNAs. Of note, alternative RNA splicing of E1 and E2 mRNAs and E6 and E7 mRNAs are of particular interest since E1 and E2, and E6 and E7 are produced from mRNAs that are produced in a mutually exclusive manner: while E1 and E6 are produced from intron-retained mRNAs, E2 and E7 are produced from the spliced variants of the same pre-mRNAs, respectively. Since E1 and E2 are functionally linked, as are E6 and E7, it is of utmost importance that the balance between production of intron-retained E1 and E6 mRNAs and spliced E2 and E7 mRNAs is optimal to meet the demand for optimal E1/E2- and E6/E7-protein ratios. A tightly tuned balance between the expression of high-risk HPV E6 and E7 oncoproteins is essential for cancer cell maintenance and malignant progression, while the balance between the expression of HPV E1 and E2 is essential during the viral replication cycle. Thus, hnRNP D may contribute to HPV16 gene expression during the viral life cycle as well as during progression to cancer and maintenance of the malignant stage of the HPV16 infected cells but that remains to be investigated.

The hnRNP D protein family consists of four members named p37, p40, p42 and p45 distinguished by the presence or absence of exon 2 and exon 7 (Figure 3A). Presence or absence of these alternatively spliced exons confers distinct biological properties to individual hnRNP D isoforms. Presence of exon 7 interrupts the C-terminal domain (CTD) of hnRNP D that has been known to affect nuclear-cytoplasmic distribution of hnRNP D (48,57). An uninterrupted CTD (p37 and p40) increased nuclear uptake, whereas an interrupted CTD (p42 and p45) resulted in cytoplasmic accumulation. Furthermore, presence of exon 7 is known to suppress ubiquitination of p42 and p45 (58). On the other hand, absence of exon 2 (p37 and p42) enhances high affinity binding to target RNAs (59). In addition, C-terminal RGG/RG motifs of hnRNP D have been shown to exert RNA chaperon and RNA annealing activities (60). Our results demonstrated that C-terminal deletions and substitutions in RGG motifs altered the subcellular distribution of hnRNP D (Figure 4C and Supplementary Figure S4B), supporting the idea that the C-terminus and/or RGG contribute to localization of hnRNP D but also revealed that neither the C-terminus nor the C-terminal RGG-motifs were essential for nuclear localization. However, the only deletion that displayed a nuclear-excluded phenotype was D8, which effectively encoded only RRM1 and RRM2 and had deletions in both N- and C-termini, suggesting that amino acid sequences in the N- and C-termini of hnRNP D collaborate to bring hnRNP D to the nucleus.

**Figure 9.**
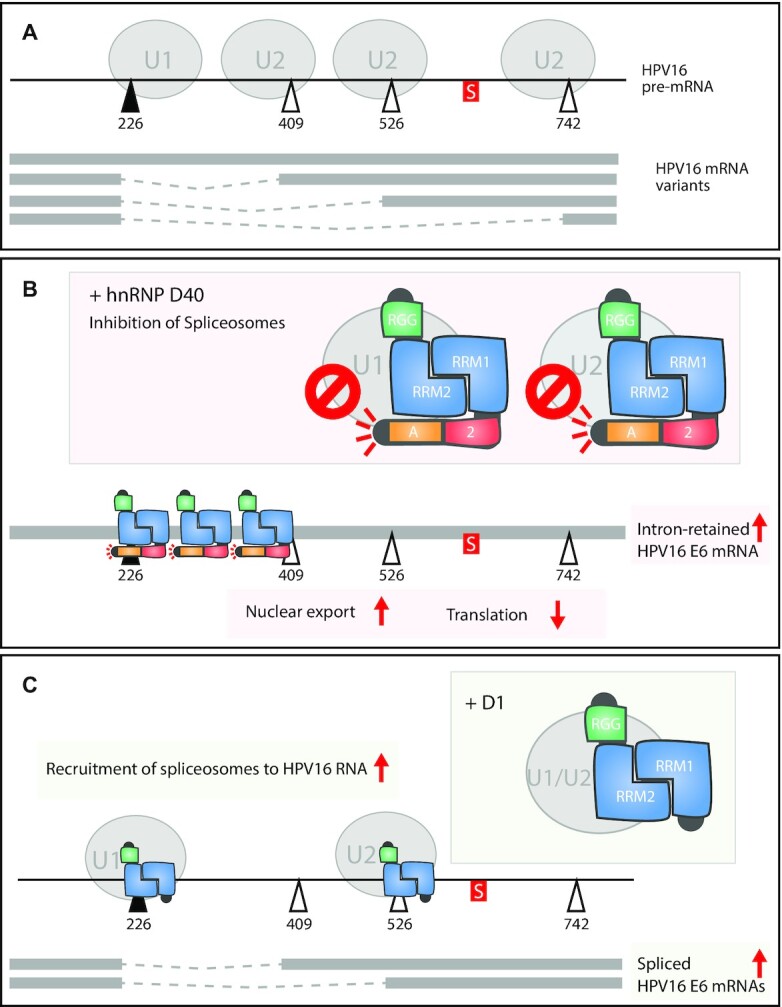
Model how hnRNP D regulates HPV16 E6/E7 mRNA splicing. (**A**) In regular condition, four splice isoforms of HPV16 E6/E7 mRNAs are produced. This is regulated by fine-tuned recruitment of cellular U1 spliceosome factor to HPV16 E6/E7 splice donor SD226 and that of U2 spliceosome factors to HPV16 E6/E7 splice acceptors SA409, SA526 or SA742. (**B**) hnRNP D40 binds to both U1 spliceosome factor and U2 spliceosome factor at its C-terminus and with some extent at its N-terminus, which inhibits spliceosome factor activity. This inhibitory function is mediated by N-terminus of hnRNP D. At the same time, hnRNP D interacts with several sites in HPV16 E6/E7 coding RNAs at its RRM1/RRM2 domain. As a result, hnRNP D mainly inhibits 226∧409 splicing on the HPV16 E6/E7 mRNAs. The intron-retained E6 mRNAs are accompanied by hnRNP D to the cytoplasm. However, hnRNP D association on intron-retained E6 mRNA suppresses translation of E6 protein by unknown mechanism. To enhance translation of HPV16 E6 protein from intron-retained E6 mRNA, hnRNP D40 must need to be replaced from intron-retained E6 mRNA. (**C**) Deletion mutant of hnRNP D40, D1 that doesn’t possess N-terminal A-rich and exon 2 but harbors intact RRM1/RRM2 domain and C-terminus, still has potential to interact with spliceosome factors and with HPV16 RNA though it loses inhibitory effect on spliceosome factors. As a result, D1 mutant mediates recruitment of spliceosome factors to HPV16 splice sites, thereby increasing HPV16 E6/E7 mRNA splicing.

All deletion mutants of hnRNP D40 displayed in this manuscript had reduced splicing inhibitory effect on HPV16 E6/E7 or E1/E2 mRNA splicing, albeit to a different extent (Figures 4 and 5). Obviously, hnRNP D40 deletion mutants D2 and D7 lost their inhibitory effect on E1/E2 mRNA splicing as well as on E6/E7 mRNA splicing (Figure 4H–K). Surprisingly, D2 appeared to activate HPV16 226∧409-splicing while D1 activated HPV16 226∧526-splicing (Figure 4D), indicating that the splicing inhibitory function of hnRNP D is located in its N-terminus and that D1 and D2 were in contact with the splicing machinery despite their N-terminal deletions. Indeed, both D1 and D2 interacted with U1-70K, U2AF65 and U2AF35, albeit at a decreased efficiency compared to wild-type hnRNP D40 (Figure 5K). In contrast to D1 that retained its ability to associate with HPV16 mRNAs, D2 displayed a reduced association with HPV16 mRNAs (Figure 5L). These results combined, we propose a model for the effect of hnRNP D on HPV16 mRNA splicing shown in Figure 9. For the interaction of hnRNP D with HPV16 RNA, both RRM1 and RRM2 domains are essential, while for the interaction with cellular spliceosome factors, U1-70K and U2AF65/35, both hnRNP D N- and C-termini regions contribute. The interaction of hnRNP D with spliceosome factors may cause functional inhibition of the splicing machinery and this inhibition appears to be mediated by hnRNP D N-terminal A-rich and exon 2-encoding region. Without the inhibitory N-terminus, hnRNP D may function as a mediator of splicing that interacts with target transcripts and recruits spliceosome factors to it, but in contrast to full-length hnRNP D, N-terminally deleted hnRNP D activates mRNA splicing. Since D1, D2 or RGG mutants differed in their inhibitory effects on HPV16 226∧526-, 226∧409- and 226∧742-RNA splicing, respectively, it appeared as if other domains than RRM1/RRM2 on hnRNP D contributed to the selection of target RNA sequences, i.e. splice site selection. Alternatively, hnRNP D40 mutants without inhibitory N-terminus (D1 and D2) functioned as decoys for full-length endogenous hnRNP D proteins and formed hetero- or homo-oligomers that may possess altered target RNA specificity or splicing inhibitory functions. In conclusion, full-length hnRNP D40 appeared to target mainly 226∧409.

In addition to the hnRNP D functional analysis, we explored hnRNP D target sites on HPV16 E6/E7 coding RNA sequence using RNA-mediated, protein pull-down assay and found that hnRNP D was associated with the E6 intronic region (nt243-445) as well as with previously identified HPV16 E6/E7 splicing silencer element located between HPV16 nt579-620 (46). In addition, hnRNP D was associated with several spots adjacent to alternative HPV16 splice sites SA526 and SA742. hnRNP D was originally identified as an RNA-binding protein that associates with the 5′- and 3′-untranslated regions of target mRNAs that contained AU-rich RNA elements (AREs) (61) to affect stability, translation or subcellular localization of target transcripts. hnRNP D/AUF1 was shown to bind directly to the AU-rich RNA elements (UAUUA) (27) and recent PAR-CLIP experiments revealed that hnRNP D/AUF1 primarily recognized U-/GU-rich sequences (e.g. UUUAGA, AGUUU, GUUUG, UUUU, UUAGUU and AGUUU) (34). These interactions affected the fate of the target transcripts in different ways, primarily by lowering steady-state levels of numerous mRNAs or by promoting translation of numerous other mRNAs. Surprisingly, hnRNP D also enhanced the steady-state levels of several target RNAs. The majority of the PAR-CLIP-identified binding sites for hnRNP D were present on multiple positions in the E6-E7 coding regions (nt97-856) and in the E1 coding region (865-2814). The previously identified HPV16 E6E7 mRNA splicing silencer UUAGAUU (46) was also found at multiple positions in the E6E7E1 coding region. This (UUAGAUU) partially overlaps with one of the binding sites for hnRNP D identified by PAR-CLIP (UUUAGA). We did not investigate if hnRNP D does bind to the multiple HPV16 RNA sequences directly, but our CLIP experiment showed that hnRNP D binds directly to HPV16 mRNAs in transfected cells. However, hnRNP D probably interacts with HPV16 mRNAs both directly and indirectly via other RNA-binding proteins. It is still unknown if each hnRNP D isoform differ in binding to specific RNA sequences. It would be of interest to use PAR-CLIP to determine potential binding sites for hnRNP D on the HPV16 mRNAs.

We showed that hnRNP D40 inhibited HPV16 early mRNA splicing and increased intron retention on 16E6 and 16E1 mRNAs and accompanied these mRNAs to the cytoplasm. This appears similar to the function of the human immunodeficiency virus (HIV) Rev protein that induces nuclear export of unspliced HIV-1 transcripts to the cytoplasm, thereby upregulating HIV Gag, Pol and Env protein levels (62–64). Here we found that hnRNP D inhibited splicing of HPV16 mRNAs and increased intron retention on HPV16 E6 and E1 mRNAs but did not enhance protein levels of either 16E6 or 16E1. As a matter of fact, the E1 and E6 protein levels were decreased in the presence of hnRNP D40. Therefore, hnRNP D may function differently from the HIV Rev protein, at least after the nuclear export of the HPV16 transcripts. It remains unclear whether hnRNP D directly affects export of intron-retained HPV16 mRNAs to the cytoplasm and it remains to be determined how these intron-retained mRNAs are exported to the cytoplasm. However, since our results demonstrate that the translation of 16E6 and 16E1 mRNA is suppressed by hnRNP D40 still sitting on the HPV16 mRNAs in the cytoplasm, we speculate that hnRNP D has a functional role in the cytoplasm. To allow E6 or E1 mRNAs to enter a productive pathway in the cytoplasm, it may be necessary to replace hnRNP D40 with unknown factor(s) on these mRNAs once they enter the cytoplasm. It is also reasonable to speculate that interactions of HPV16 mRNAs with hnRNP D affects the half-life of the intron-retained HPV16 mRNAs but that remains to be determined. To uncover the fate of the intron-retained HPV16 E1 and E6 mRNAs in the cytoplasm, a better understanding of hnRNP D and its role in the cytoplasm is needed.

Interactions between hnRNP D and various viral RNAs have been described previously, both with nuclear-restricted viral RNAs such as those produced by Epstein-Barr virus (EBV) (65) and cytoplasm-restricted viral RNAs such as those produced by viruses of the Picornaviridae family, including poliovirus, Coxsackievirus B3 (CVB3), Human Rhinovirus (HRV) and Enterovirus EV71 (66,67) or of the Flaviviridae family, including West Nile virus (WNV) and Dengue virus (DENV) (68,69), as well as with HIV of the Retroviridae family (70). The consequence of the interactions between hnRNP D and the various viral RNAs differs. Interactions of hnRNP D and EBV EBER1 noncoding RNAs are believed to modulate cellular alternative RNA splicing, although direct evidence is lacking at this point (65). Interactions between hnRNP D and Picornaviral genomic RNAs at the internal ribosome entry site (IRES) of the 5′-non-coding region (NCR) or at the 3′-NCR, resulted in inhibition of translation (71) or decreased viral genomic RNA levels (67), respectively. Interactions between hnRNP D and flaviviral genomic RNAs, either at the 5′-NCR or at the 3′-NCR contributed to a shift in the RNA structure from a linear to a cyclic viral genome of which the latter is favored as a template for viral RNA replication rather than translation (68,69). Interactions between different hnRNP D isoforms and HIV-1 exon 3-RNA altered the relative levels of spliced and unspliced HIV-1 mRNAs (72). In conclusion, hnRNP D plays a major role in replication and gene expression of multiple viruses, including DNA viruses and RNA viruses, affecting nuclear as well as cytoplasmic functions of these viral RNAs. In particular, hnRNP D appears to inhibit translation of mRNAs produced by several different viruses, which is in line with our results presented here.

In contrast to the RNA viruses described above that replicate to high levels and cause acute infections, HPVs establish persistent infections that normally last for 12–24 months, thereby requiring elaborate immune evasion mechanisms, suggesting that the inhibitory effect of hnRNP D on the translation of the E1 and E6 mRNAs may contribute to persistence in the presence of a functional immune system of the host. At the regulatory level, the ratio of E1 to E2, produced from intron-retained and spliced mRNAs respectively, may affect various steps in the HPV life cycle. While higher levels of E1 than E2 is required for genomic DNA replication, increased E2 levels will eventually shut down the early HPV promoter to allow for a re-entry into the cellular differentiation program and completion of the HPV life cycle. Regarding E6 and E7 proteins that are produced from intron-retained and spliced mRNAs respectively, an optimal ratio of E6 and E7 is required to achieve an optimal balance between antiapoptotic and promitotic functions exerted by E6 and E7, respectively. Although it remains to be determined how the intron-retained HPV E1 and E6 mRNAs are translated, hnRNP D may contribute to the modulation of HPV16 mRNA translation and to the control of the delicate balance of the E1/E2- and E6/E7 protein ratios during HPV16 infection.

Finally and importantly, the inhibitory effect of hnRNP D40 on HPV16 E6 or E1 mRNA splicing was reproduced with episomal genomic HPV16 DNA in human primary keratinocytes. This is of significance since the HPV16 genome normally exists as an episome during its life cycle in infected cells *in vivo*. Furthermore, we demonstrated an association between endogenous HPV16 RNA and hnRNP D proteins in HN26 cells, a cell line recently isolated from a male patient with HPV16-positive tonsillar cancer, and in the previously described HPV16-immortalized human keratinocyte cell line 3310 (Figure 8). Interestingly, induction of keratinocyte differentiation in primary keratinoytes appeared to promote production of intron-retained E1 mRNAs, an effect that was enhanced by overexpression of hnRNP D40, further supporting a role for hnRNP D in the differentiation-dependent HPV16 gene expression program. The hnRNP D proteins are expressed in cervical epithelium as well as in tonsillar epithelial cells *in vivo* and appears to be overexpressed in cervical and tonsillar cancer (https://www.proteinatlas.org/ENSG00000138668-HNRNPD), indicating that hnRNP D is available to HPV16 in its natural environment as well as during progression to cancer. It has been shown that the levels of multiple cellular RNA-binding proteins are altered or differ in functional activity through cervical cancer development caused by HPV infection (73), underscoring the importance of RNA-binding proteins during progression of HPV-infected cells to cancer. In fact, altered expression of hnRNP D protein has been observed in different cancers (74) supporting the idea of a role for hnRNP D in carcinogenesis. Modulation of HPV16 gene expression by hnRNP D suggests that hnRNP D may be a future target for anti-HPV and anti-cancer treatment. This conclusion is reinforced by our results presented here, demonstrating that knockdown of hnRNP D in HPV16-driven cervical cancer cells enhanced production of the HPV16 E7 oncoprotein.

## Supplementary Material

gkac132_Supplemental_File

## References

[1] Chow L.T. , Broker T.R., Steinberg B.M. The natural history of human papillomavirus infections of the mucosal epithelia. APMIS. 2010; 118:422–449. 20553526 10.1111/j.1600-0463.2010.02625.x

[2] McBride A.A. Human papillomaviruses: diversity, infection and host interactions. Nat. Rev. Microbiol. 2021; 20:95–108. 34522050 10.1038/s41579-021-00617-5

[3] Bouvard V. , Baan R., Straif K., Grosse Y., Secretan B., El Ghissassi F., Benbrahim-Tallaa L., Guha N., Freeman C., Galichet L. et al. A review of human carcinogens–Part B: biological agents. Lancet Oncol. 2009; 10:321–322. 19350698 10.1016/s1470-2045(09)70096-8

[4] Moody C.A. , Laimins L.A. Human papillomavirus oncoproteins: pathways to transformation. Nat. Rev. Cancer. 2010; 10:550–560. 20592731 10.1038/nrc2886

[5] Akagi K. , Li J., Broutian T.R., Padilla-Nash H., Xiao W., Jiang B., Rocco J.W., Teknos T.N., Kumar B., Wangsa D. et al. Genome-wide analysis of HPV integration in human cancers reveals recurrent, focal genomic instability. Genome Res. 2014; 24:185–199. 24201445 10.1101/gr.164806.113 PMC3912410

[6] Martinez-Zapien D. , Ruiz F.X., Poirson J., Mitschler A., Ramirez J., Forster A., Cousido-Siah A., Masson M., Vande Pol S., Podjarny A. et al. Structure of the E6/E6AP/p53 complex required for HPV-mediated degradation of p53. Nature. 2016; 529:541–545. 26789255 10.1038/nature16481 PMC4853763

[7] Mantovani F. , Banks L. The human papillomavirus E6 protein and its contribution to malignant progression. Oncogene. 2001; 20:7874–7887. 11753670 10.1038/sj.onc.1204869

[8] James M.A. , Lee J.H., Klingelhutz A.J. Human papillomavirus type 16 E6 activates NF-kappaB, induces cIAP-2 expression, and protects against apoptosis in a PDZ binding motif-dependent manner. J. Virol. 2006; 80:5301–5307. 16699010 10.1128/JVI.01942-05 PMC1472131

[9] White E.A. , Kramer R.E., Tan M.J., Hayes S.D., Harper J.W., Howley P.M. Comprehensive analysis of host cellular interactions with human papillomavirus E6 proteins identifies new E6 binding partners and reflects viral diversity. J. Virol. 2012; 86:13174–13186. 23015706 10.1128/JVI.02172-12 PMC3503137

[10] Bergvall M. , Melendy T., Archambault J. The E1 proteins. Virology. 2013; 445:35–56. 24029589 10.1016/j.virol.2013.07.020 PMC3811109

[11] McBride A.A. The papillomavirus E2 proteins. Virology. 2013; 445:57–79. 23849793 10.1016/j.virol.2013.06.006 PMC3783563

[12] Burley M. , Roberts S., Parish J.L. Epigenetic regulation of human papillomavirus transcription in the productive virus life cycle. Semin. Immunopathol. 2020; 42:159–171. 31919577 10.1007/s00281-019-00773-0 PMC7174255

[13] Kurg R. , Uusen P., Võsa L., Ustav M. Human papillomavirus E2 protein with single activation domain initiates HPV18 genome replication, but is not sufficient for long-term maintenance of virus genome. Virology. 2010; 408:159–166. 20940072 10.1016/j.virol.2010.09.010

[14] Kadaja M. , Isok-Paas H., Laos T., Ustav E., Ustav M. Mechanism of genomic instability in cells infected with the high-risk human papillomaviruses. PLoS Pathog. 2009; 5:e1000397. 19390600 10.1371/journal.ppat.1000397 PMC2666264

[15] DiMaio D. , Petti L.M. The E5 proteins. Virology. 2013; 445:99–114. 23731971 10.1016/j.virol.2013.05.006 PMC3772959

[16] Doorbar J. The E4 protein; structure, function and patterns of expression. Virology. 2013; 445:80–98. 24016539 10.1016/j.virol.2013.07.008

[17] Johansson C. , Schwartz S. Regulation of human papillomavirus gene expression by splicing and polyadenylation. Nat. Rev. Microbiol. 2013; 11:239–251. 23474685 10.1038/nrmicro2984

[18] Kajitani N. , Schwartz S. Role of Viral Ribonucleoproteins in Human Papillomavirus Type 16 Gene Expression. Viruses. 2020; 12:1110. 10.3390/v12101110 PMC7600041 33007936

[19] Ajiro M. , Zheng Z.M. Oncogenes and RNA splicing of human tumor viruses. Emerg. Microbes Infect. 2014; 3:e63. 26038756 10.1038/emi.2014.62 PMC4185361

[20] Zheng Z.M. Viral oncogenes, noncoding RNAs, and RNA splicing in human tumor viruses. Int. J. Biol. Sci. 2010; 6:730–755. 21152115 10.7150/ijbs.6.730 PMC2999850

[21] Graham S.V. , Faizo A.A.A. Control of human papillomavirus gene expression by alternative splicing. Virus Res. 2017; 231:83–95. 27867028 10.1016/j.virusres.2016.11.016 PMC5335905

[22] Graham S.V. Keratinocyte differentiation-dependent Human Papillomavirus gene regulation. Viruses. 2017; 9:245. 10.3390/v9090245 PMC5618011 28867768

[23] Fay J. , Kelehan P., Lambkin H., Schwartz S. Increased expression of cellular RNA-binding proteins in HPV-induced neoplasia and cervical cancer. J. Med. Virol. 2009; 81:897–907. 19319956 10.1002/jmv.21406

[24] Mole S. , McFarlane M., Chuen-Im T., Milligan S.G., Millan D., Graham S.V. RNA splicing factors regulated by HPV16 during cervical tumour progression. J. Pathol. 2009; 219:383–391. 19718710 10.1002/path.2608 PMC2779514

[25] Dreyfuss G. , Kim V.N., Kataoka N. Messenger-RNA-binding proteins and the messages they carry. Nat. Rev. Mol. Cell Biol. 2002; 3:195–205. 11994740 10.1038/nrm760

[26] Chen M. , Manley J.L. Mechanisms of alternative splicing regulation: insights from molecular and genomics approaches. Nat. Rev. Mol. Cell Biol. 2009; 10:741–754. 19773805 10.1038/nrm2777 PMC2958924

[27] Van Nostrand E.L. , Freese P., Pratt G.A., Wang X., Wei X., Xiao R., Blue S.M., Chen J.Y., Cody N.A.L., Dominguez D. et al. A large-scale binding and functional map of human RNA-binding proteins. Nature. 2020; 583:711–719. 32728246 10.1038/s41586-020-2077-3 PMC7410833

[28] Geuens T. , Bouhy D., Timmerman V. The hnRNP family: insights into their role in health and disease. Hum. Genet. 2016; 135:851–867. 27215579 10.1007/s00439-016-1683-5 PMC4947485

[29] Cartegni L. , Maconi M., Morandi E., Cobianchi F., Riva S., Biamonti G. hnRNP A1 selectively interacts through its Gly-rich domain with different RNA-binding proteins. J. Mol. Biol. 1996; 259:337–348. 8676373 10.1006/jmbi.1996.0324

[30] Jankowsky E. , Harris M.E. Specificity and nonspecificity in RNA-protein interactions. Nat. Rev. Mol. Cell Biol. 2015; 16:533–544. 26285679 10.1038/nrm4032 PMC4744649

[31] Gratacós F.M. , Brewer G. The role of AUF1 in regulated mRNA decay. Wiley Interdiscip. Rev. RNA. 2010; 1:457–473. 21956942 10.1002/wrna.26 PMC3608466

[32] Abdelmohsen K. , Tominaga-Yamanaka K., Srikantan S., Yoon J.H., Kang M.J., Gorospe M. RNA-binding protein AUF1 represses Dicer expression. Nucleic Acids Res. 2012; 40:11531–11544. 23066106 10.1093/nar/gks930 PMC3526313

[33] Liao B. , Hu Y., Brewer G. Competitive binding of AUF1 and TIAR to MYC mRNA controls its translation. Nat. Struct. Mol. Biol. 2007; 14:511–518. 17486099 10.1038/nsmb1249

[34] Yoon J.H. , De S., Srikantan S., Abdelmohsen K., Grammatikakis I., Kim J., Kim K.M., Noh J.H., White E.J., Martindale J.L. et al. PAR-CLIP analysis uncovers AUF1 impact on target RNA fate and genome integrity. Nat. Commun. 2014; 5:5248. 25366541 10.1038/ncomms6248 PMC4291169

[35] Eversole A. , Maizels N. In vitro properties of the conserved mammalian protein hnRNP D suggest a role in telomere maintenance. Mol. Cell. Biol. 2000; 20:5425–5432. 10891483 10.1128/mcb.20.15.5425-5432.2000 PMC85994

[36] Pont A.R. , Sadri N., Hsiao S.J., Smith S., Schneider R.J. mRNA decay factor AUF1 maintains normal aging, telomere maintenance, and suppression of senescence by activation of telomerase transcription. Mol. Cell. 2012; 47:5–15. 22633954 10.1016/j.molcel.2012.04.019 PMC3966316

[37] Torrisani J. , Unterberger A., Tendulkar S.R., Shikimi K., Szyf M. AUF1 cell cycle variations define genomic DNA methylation by regulation of DNMT1 mRNA stability. Mol. Cell. Biol. 2007; 27:395–410. 17030625 10.1128/MCB.01236-06 PMC1800664

[38] Lapucci A. , Donnini M., Papucci L., Witort E., Tempestini A., Bevilacqua A., Nicolin A., Brewer G., Schiavone N., Capaccioli S. AUF1 Is a bcl-2 A + U-rich element-binding protein involved in bcl-2 mRNA destabilization during apoptosis. J. Biol. Chem. 2002; 277:16139–16146. 11856759 10.1074/jbc.M201377200

[39] Lu J.Y. , Sadri N., Schneider R.J. Endotoxic shock in AUF1 knockout mice mediated by failure to degrade proinflammatory cytokine mRNAs. Genes Dev. 2006; 20:3174–3184. 17085481 10.1101/gad.1467606 PMC1635151

[40] Kemmerer K. , Fischer S., Weigand J.E. Auto- and cross-regulation of the hnRNPs D and DL. RNA. 2018; 24:324–331. 29263134 10.1261/rna.063420.117 PMC5824352

[41] Fragkouli A. , Koukouraki P., Vlachos I.S., Paraskevopoulou M.D., Hatzigeorgiou A.G., Doxakis E. Neuronal ELAVL proteins utilize AUF-1 as a co-partner to induce neuron-specific alternative splicing of APP. Sci. Rep. 2017; 7:44507. 28291226 10.1038/srep44507 PMC5349543

[42] Li X. , Johansson C., Cardoso Palacios C., Mossberg A., Dhanjal S., Bergvall M., Schwartz S. Eight nucleotide substitutions inhibit splicing to HPV-16 3′-splice site SA3358 and reduce the efficiency by which HPV-16 increases the life span of primary human keratinocytes. PLoS One. 2013; 8:e72776. 24039800 10.1371/journal.pone.0072776 PMC3767658

[43] Li X. , Johansson C., Glahder J., Mossberg A.K., Schwartz S. Suppression of HPV-16 late L1 5′-splice site SD3632 by binding of hnRNP D proteins and hnRNP A2/B1 to upstream AUAGUA RNA motifs. Nucleic Acids Res. 2013; 41:10488–10508. 24013563 10.1093/nar/gkt803 PMC3905901

[44] Wu C. , Nilsson K., Zheng Y., Ekenstierna C., Sugiyama N., Forslund O., Kajitani N., Yu H., Wennerberg J., Ekblad L. et al. Short half-life of HPV16 E6 and E7 mRNAs sensitizes HPV16-positive tonsillar cancer cell line HN26 to DNA-damaging drugs. Int. J. Cancer. 2019; 144:297–310. 30303514 10.1002/ijc.31918 PMC6587446

[45] Somberg M. , Zhao X., Fröhlich M., Evander M., Schwartz S. Polypyrimidine tract binding protein induces human papillomavirus type 16 late gene expression by interfering with splicing inhibitory elements at the major late 5′ splice site, SD3632. J. Virol. 2008; 82:3665–3678. 18216120 10.1128/JVI.02140-07 PMC2268445

[46] Zheng Y. , Jönsson J., Hao C., Shoja Chaghervand S., Cui X., Kajitani N., Gong L., Wu C., Schwartz S. Heterogeneous nuclear ribonucleoprotein A1 (hnRNP A1) and hnRNP A2 Inhibit Splicing to Human Papillomavirus 16 Splice Site SA409 through a UAG-containing sequence in the E7 coding region. J. Virol. 2020; 94:e01509-20. 32759322 10.1128/JVI.01509-20 PMC7527060

[47] Dhanjal S. , Kajitani N., Glahder J., Mossberg A.K., Johansson C., Schwartz S. Heterogeneous nuclear ribonucleoprotein C proteins interact with the Human Papillomavirus Type 16 (HPV16) early 3′-untranslated region and alleviate suppression of HPV16 Late L1 mRNA splicing. J. Biol. Chem. 2015; 290:13354–13371. 25878250 10.1074/jbc.M115.638098 PMC4505585

[48] Sarkar B. , Lu J.Y., Schneider R.J. Nuclear import and export functions in the different isoforms of the AUF1/heterogeneous nuclear ribonucleoprotein protein family. J. Biol. Chem. 2003; 278:20700–20707. 12668672 10.1074/jbc.M301176200

[49] Nasim M.T. , Chernova T.K., Chowdhury H.M., Yue B.G., Eperon I.C. HnRNP G and Tra2beta: opposite effects on splicing matched by antagonism in RNA binding. Hum. Mol. Genet. 2003; 12:1337–1348. 12761049 10.1093/hmg/ddg136

[50] Guang S. , Felthauser A.M., Mertz J.E. Binding of hnRNP L to the pre-mRNA processing enhancer of the herpes simplex virus thymidine kinase gene enhances both polyadenylation and nucleocytoplasmic export of intronless mRNAs. Mol. Cell. Biol. 2005; 25:6303–6313. 16024770 10.1128/MCB.25.15.6303-6313.2005 PMC1190326

[51] Huang J. , Li S.J., Chen X.H., Han Y., Xu P. hnRNP-R regulates the PMA-induced c-fos expression in retinal cells. Cell. Mol. Biol. Lett. 2008; 13:303–311. 18197392 10.2478/s11658-008-0002-0 PMC6275800

[52] Chou M.Y. , Rooke N., Turck C.W., Black D.L. hnRNP H is a component of a splicing enhancer complex that activates a c-src alternative exon in neuronal cells. Mol. Cell. Biol. 1999; 19:69–77. 9858532 10.1128/mcb.19.1.69 PMC83866

[53] Collier B. , Oberg D., Zhao X., Schwartz S. Specific inactivation of inhibitory sequences in the 5′ end of the human papillomavirus type 16 L1 open reading frame results in production of high levels of L1 protein in human epithelial cells. J. Virol. 2002; 76:2739–2752. 11861841 10.1128/JVI.76.6.2739-2752.2002 PMC135970

[54] Busch A. , Hertel K.J. Evolution of SR protein and hnRNP splicing regulatory factors. Wiley Interdiscip. Rev. RNA. 2012; 3:1–12. 21898828 10.1002/wrna.100 PMC3235224

[55] Forslund O. , Sugiyama N., Wu C., Ravi N., Jin Y., Swoboda S., Andersson F., Bzhalava D., Hultin E., Paulsson K. et al. A novel human in vitro papillomavirus type 16 positive tonsil cancer cell line with high sensitivity to radiation and cisplatin. BMC Cancer. 2019; 19:265. 30909875 10.1186/s12885-019-5469-8 PMC6434888

[56] Johansson C. , Jamal Fattah T., Yu H., Nygren J., Mossberg A.K., Schwartz S. Acetylation of intragenic histones on HPV16 correlates with enhanced HPV16 gene expression. Virology. 2015; 482:244–259. 25900886 10.1016/j.virol.2015.02.053

[57] Arao Y. , Kuriyama R., Kayama F., Kato S. A nuclear matrix-associated factor, SAF-B, interacts with specific isoforms of AUF1/hnRNP D. Arch. Biochem. Biophys. 2000; 380:228–236. 10933876 10.1006/abbi.2000.1938

[58] Laroia G. , Schneider R.J. Alternate exon insertion controls selective ubiquitination and degradation of different AUF1 protein isoforms. Nucleic Acids Res. 2002; 30:3052–3058. 12136087 10.1093/nar/gkf444 PMC135764

[59] Wagner B.J. , DeMaria C.T., Sun Y., Wilson G.M., Brewer G. Structure and genomic organization of the human AUF1 gene: alternative pre-mRNA splicing generates four protein isoforms. Genomics. 1998; 48:195–202. 9521873 10.1006/geno.1997.5142

[60] Meyer A. , Golbik R.P., Sänger L., Schmidt T., Behrens S.E., Friedrich S. The RGG/RG motif of AUF1 isoform p45 is a key modulator of the protein's RNA chaperone and RNA annealing activities. RNA Biol. 2019; 16:960–971. 30951406 10.1080/15476286.2019.1602438 PMC6550787

[61] Zhang W. , Wagner B.J., Ehrenman K., Schaefer A.W., DeMaria C.T., Crater D., DeHaven K., Long L., Brewer G. Purification, characterization, and cDNA cloning of an AU-rich element RNA-binding protein, AUF1. Mol. Cell. Biol. 1993; 13:7652–7665. 8246982 10.1128/mcb.13.12.7652-7665.1993 PMC364837

[62] Felber B.K. , Hadzopoulou-Cladaras M., Cladaras C., Copeland T., Pavlakis G.N. rev protein of human immunodeficiency virus type 1 affects the stability and transport of the viral mRNA. Proc. Natl. Acad. Sci. U.S.A. 1989; 86:1495–1499. 2784208 10.1073/pnas.86.5.1495 PMC286723

[63] Felber B.K. , Zolotukhin A.S., Pavlakis G.N. Posttranscriptional control of HIV-1 and other retroviruses and its practical applications. Adv. Pharmacol. 2007; 55:161–197. 17586315 10.1016/S1054-3589(07)55005-2

[64] Ostermann P.N. , Ritchie A., Ptok J., Schaal H. Let It Go: HIV-1. J. Virol. 2021; 95:e0034221. 33980600 10.1128/JVI.00342-21 PMC8274598

[65] Lee N. , Pimienta G., Steitz J.A. AUF1/hnRNP D is a novel protein partner of the EBER1 noncoding RNA of Epstein-Barr virus. RNA. 2012; 18:2073–2082. 23012480 10.1261/rna.034900.112 PMC3479396

[66] Cathcart A.L. , Semler B.L. Differential restriction patterns of mRNA decay factor AUF1 during picornavirus infections. J. Gen. Virol. 2014; 95:1488–1492. 24722678 10.1099/vir.0.064501-0 PMC4059269

[67] Wong J. , Si X., Angeles A., Zhang J., Shi J., Fung G., Jagdeo J., Wang T., Zhong Z., Jan E. et al. Cytoplasmic redistribution and cleavage of AUF1 during coxsackievirus infection enhance the stability of its viral genome. FASEB J. 2013; 27:2777–2787. 23572232 10.1096/fj.12-226498

[68] Friedrich S. , Schmidt T., Geissler R., Lilie H., Chabierski S., Ulbert S., Liebert U.G., Golbik R.P., Behrens S.E. AUF1 p45 promotes West Nile virus replication by an RNA chaperone activity that supports cyclization of the viral genome. J. Virol. 2014; 88:11586–11599. 25078689 10.1128/JVI.01283-14 PMC4178777

[69] Friedrich S. , Engelmann S., Schmidt T., Szczepankiewicz G., Bergs S., Liebert U.G., Kümmerer B.M., Golbik R.P., Behrens S.E. The host factor AUF1 p45 supports flavivirus propagation by triggering the RNA switch required for viral genome cyclization. J. Virol. 2018; 92:e01647-17. 29263261 10.1128/JVI.01647-17 PMC5827407

[70] Lund N. , Milev M.P., Wong R., Sanmuganantham T., Woolaway K., Chabot B., Abou Elela S., Mouland A.J., Cochrane A. Differential effects of hnRNP D/AUF1 isoforms on HIV-1 gene expression. Nucleic Acids Res. 2012; 40:3663–3675. 22187150 10.1093/nar/gkr1238 PMC3333888

[71] Ullmer W. , Semler B.L. Direct and indirect effects on viral translation and RNA replication are required for AUF1 restriction of enterovirus infections in human cells. mBio. 2018; 9:e01669-18. 30181254 10.1128/mBio.01669-18 PMC6123441

[72] Hillebrand F. , Peter J.O., Brillen A.L., Otte M., Schaal H., Erkelenz S. Differential hnRNP D isoform incorporation may confer plasticity to the ESSV-mediated repressive state across HIV-1 exon 3. Biochim. Biophys. Acta Gene Regul. Mech. 2017; 1860:205–217. 27919832 10.1016/j.bbagrm.2016.12.001

[73] Cerasuolo A. , Buonaguro L., Buonaguro F.M., Tornesello M.L. The role of RNA splicing factors in cancer: regulation of viral and human gene expression in Human Papillomavirus-related cervical cancer. Front. Cell Dev. Biol. 2020; 8:474. 32596243 10.3389/fcell.2020.00474 PMC7303290

[74] Zucconi B.E. , Wilson G.M. Modulation of neoplastic gene regulatory pathways by the RNA-binding factor AUF1. Front. Biosci. (Landmark Ed.). 2011; 16:2307–2325. 21622178 10.2741/3855 PMC3589912

