# Supplementary material for: HnRNP D activates production of HPV16 E1 and E6 mRNAs by promoting intron retention
Source: Nucleic Acids Res. 2022 Mar 2;50(5):2782–806. doi: 10.1093/nar/gkac132 (PMC8934624; doi:10.1093/nar/gkac132)
Supplement: gkac132_Supplemental_File [file gkac132_supplemental_file.pdf]

SUPPLEMENTARY FIGURES LEGENDS

Supplementary Figure S1

(A) Schematic structures of pC97ELsLuc and representative HPV16 early transcripts produced from pC97ELsLuc and spliced or not spliced at SD880. Plasmid pC97ELsLuc encodes all HPV16 genes. Early promoter P97 was replaced by CMV promoter. Secreted luciferase (sLuc) gene was integrated in the L1 gene following the poliovirus 2A internal ribosomal entry site (IRES) sequence. Numbers refer to the HPV16 reference strain GeneBank: K02718.1. Early and late genes are indicated. Black oval: splice donor. White oval: splice acceptor. pAE: HPV16 early polyadenylation site. pAL: HPV16 late polyadenylation site. Arrows indicate primer annealing positions. **(B)** Indicated hnRNP D isoform-encoding plasmids were cotransfected with pC97ELsLuc into HeLa cells. RNA was extracted and subjected to RT-PCR with primers 97S and E1AS. This RT-PCR reaction detected mRNAs that were unspliced at SD880. The authenticity of intron-retained E6E7E1 mRNA band (intron-retained, IR) was determined by sequencing and confirmed by the reverse transcriptase negative control assay on the same RNA samples. **(C-E)** Dose-dependent effect of hnRNP D40 was monitored by cotransfecting pC97ELsLuc with serially diluted hnRNP D40 encoding plasmid. RNA was extracted and subjected to RT-PCR with HPV16 RT-PCR primers 97S+880AS (**C**), 773S+E2AS (**D**) or 773S+E4AS (**E**). (**F**) The four mRNAs isoforms of hnRNP D in indicated cell lines were analyzed by RT-PCR. (**G**) Exogeneous expression of hnRNP D40 (lane 3) was determined to compare with endogeneous hnRNP D (lane 1). (**H**) The exogenous expression of each isoform of hnRNP D protein in HeLa cells were analyzed by Western-blotting using anti-FLAG antibody.

Supplementary Figure S2

Determination of the consistent hnRNP D effect on HPV16 early mRNA splicing both from pC97ELsLuc and pBELsLuc. (A and B) Schematic representation of HPV16 subgenomic plasmid pC97ELsLuc, pBELsLuc and representative HPV16 transcripts investigated in (C) and (D) are shown. Plasmid pC97ELsLuc encodes all HPV16 genes. Early promoter P97 was replaced by human cytomegalovirus immediate early promoter (CMV). Plasmid pBELsLuc encodes all HPV16 genes except E6 and E7 and is driven by the CMV promoter. Secreted luciferase (sLuc) gene was integrated in the L1 gene following the poliovirus 2A internal ribosomal entry site (IRES) sequence. Early and late genes are indicated. Black oval: splice donor. White oval: splice acceptor. pAE: HPV16 early polyadenylation site. pAL: HPV16 late polyadenylation site. Numbers refer to the HPV16 reference strain GeneBank: K02718.1. Schematic representation of HPV16 alternatively spliced mRNAs produced from pBELsLuc are shown below pBELsLuc. Arrows indicate annealing positions of HPV16 RT-PCR primers. **(C**) hnRNP D37 or hnRNP D40 encoding plasmid was cotransfected with pC97ELsLuc or pBELsluc, RNA was extracted and HPV16 E1/E2 mRNA splicing or E4 mRNA splicing was determined by RT-PCR with primer pairs 773S+E2AS or 773S+E4AS, respectively. **(D)** Serially diluted hnRNP D40 plasmid was cotransfected with pC97ELsLuc or pBELsLuc. RNA was extracted and HPV16 E1/E2 mRNA splicing was determined by RT-PCR with primer pair 773S+E2AS. intron-retained (IR) E1 mRNAs and spliced 880^2709 mRNAs are indicated.

Supplementary Figure S3

Subcellular localization of hnRNP D40 and hnRNP D40-mutants fused to EGFP. (A) Higher magnifications of microscopic images adapted from **Fig. 4C**. A cell highlighted by white arrowhead in each image is the same cell that is highlighted in **Fig. 4C**. Enhanced green fluorescent protein gene was fused in frame with the entire hnRNP D40 open reading frame or mutants thereof as indicated. EGFP(-), plasmid expressing EGFP not fused to any protein. **(B and C)** Proportion of each alternatively spliced HPV16 E6E7 mRNA isoforms (intron-retained E6, 226^409, 226^526 and 226^742) or E1E2 mRNA isoforms (intron-retained E1, 880^2582 and 880^2709) over all spliced isoforms displayed in **Fig. 3D or 3E**. The proportion of a spliced isoform was calculated as a percentage of an isoform band intensity over the sum of all spliced isoform band intensities in each lane. Percentage bar graphs of all isoforms were built with mean values of three independent replicates. (**D**) Longer exposures of gel images of HPV16 E1E2 mRNA RT-PCR in **Fig. 3D and E** (773S+E2QAS) to enhance detection of the longer and inefficiently amplified cDNAs representing intron-retained E1 mRNAs.

Supplementary Figure S4

(A) hnRNP D40 and hnRNP D40 mutants had similar effect on HPV16 mRNA splicing in 293T cells (upper panel) and HeLa cells (lower panel). Plasmids expressing hnRNP D40 or the indicated hnRNP D40-mutants were individually cotransfected with pC97ELsLuc into 293T cells or HeLa cells, RNA was extracted and HPV16 E6E7 mRNA-splicing was analyzed by RT-PCR with HPV16 RT-PCR primers 97S+880AS. GAPDH, gapdh RT-PCR with primers GAPDHF and GAPDHR. (**B)** Subcellular localization of the EGFP-AGG mutant that in which "R" in the RG/RGG-motifs is replaced by "A" as described in **Fig. 5A**. Higher magnification image of EGFP-AGG was shown as "EGFP-AGG (H)". The same cells were highlighted with white arrowhead. (**C**) Proportion of the indicated spliced isoforms of HPV16 mRNAs quantitated from **Fig. 5I and J**. Percentage of the indicated spliced isoform over the sum of all spliced isoforms in each lane was calculated as described in **Fig. 4H-K**. Significance was calculated as described in Materials and Methods. **(D)** The coimmunoprecipitation of hnRNP D40 protein and SF3b or PABP-C1 analyzed as described in Figure 5K.

Supplementary Figure S5

(A) HPV16 subgenomic plasmid pC97ELsLuc encodes all HPV16 genes. Early promoter P97 was replaced by human cytomegalovirus immediate early promoter (CMV). Secreted luciferase (sLuc) gene was integrated in L1 gene following the poliovirus 2A internal ribosomal entry site (IRES) sequence. Early and late genes are indicated. Black opal: splice donor. White opal: splice acceptor. pAE: HPV16 early polyadenylation site. pAL: HPV16 late polyadenylation site. Numbers refer to the HPV16 reference strain GeneBank: K02718.1. "S" in red box represents a previously identified splicing silencer. **(B-E)** Schematic representations of HPV16 subgenomic expression plasmids pX656 **(B)**, pX478 **(C)**, pXH856F **(D)** and pXH856SDmF **(E)** used in **Figure 6**. HA-tags and FLAG-tags fused to HPV16 E6 and E7, respectively, in pXH856F and pX856SDmF are indicated. Wild type SD226 (GAG|GUAUAUGA : a

vertical line indicates 5' splice site) in pXH856F was changed to (GAG|GCCUAUGA: underline indicates changed nucleotide) for SD226 inactivation in pXH856SDmF. RT-PCR primers are indicated. The various alternatively spliced mRNAs produced by these plasmids are indicated. CMV: CMV promoter.

Supplementary Figure S6

Quality controls of nuclear/cytoplasmic fractionation for Figure 7A by Western-blotting analysis using anti-Lamin B, anti-SRSF1 or anti-SRSF2 antibody.

Supplementary Figure S7

(A) HPV16 subgenomic plasmid pBELsLuc encodes all HPV16 genes except E6 and E7 and is driven by the CMV promoter. Numbers refer to the HPV16 reference strain GeneBank: K02718.1. Early and late genes are indicated. Black oval: splice donor. White oval: splice acceptor. pAE: HPV16 early polyadenylation site. pAL: HPV16 late polyadenylation site. Secreted luciferase (sLuc) gene was integrated in L1 gene following the poliovirus 2A internal ribosomal entry site (IRES) sequence. (**B and C**) Schematic drawings of HPV16 subgenomic plasmids pBELEN and pBELENdE1 of which the latter harbors a deletion inside the E1 gene (Δnt1150-2481). Arrows indicate RT-PCR primer annealing positions. (**D**) Effect of hnRNP D40 overexpression on HPV16 E1/E2 mRNA splicing using HPV16 reporter plasmid pBELEN. pBELEN was transfected into HeLa cells in the absence (-) or presence (+) of hnRNP D40 expression plasmid. RNA was extracted and RT-PCR was performed with HPV16 primers 773S+E2Xba. intron-retained E1 mRNAs and spliced 880^2709 mRNAs are indicated. (**E**) HPV16 subgenomic plasmid pBELEN was transfected into HeLa cells in the absence (-) or presence (+) of hnRNP D40 expression plasmid. The transfected cells were fractionated into nuclear and cytoplasmic fractions, RNA was extracted and RT-PCR was performed with HPV16 primers 773S+E2Xba. intron-retained E1 mRNAs and spliced 880^2709 mRNAs are indicated. Unspliced and spliced actin mRNAs were detected by RT-PCR to control for subcellular fractionation. N, nuclear fraction; C, cytoplasmic fraction. **(F)** Quantitation of the RT-PCR products. The percentage of cytoplasmic intron-retained E1 mRNA over the sum of cytoplasmic and nuclear levels were calculated as described in **Fig. 6D**. (**G and H**) Schematic representations of HPV16 subgenomic plasmids p16E1-3xF and p16E1SDm-3xF encoding HPV16 E1 tagged with three FLAG-tags. Wild type SD880 (CAG|GUACCA: a vertical line indicates 5' splice site) and SD1302 (CAG|GUAGAA) in p16E1-3xF were changed to (CAG|CUACCA: underline indicates a changed nucleotide) for SD880 inactivation or (CAG|CUAGAA) for SD1302 inactivation in p16E1SDm-3xF, respectively. Numbers refer to the HPV16 reference strain GeneBank: K02718.1. Black oval: splice donor. White oval: splice acceptor. "x" represents mutational inactivation of HPV16 5'-splice sites SD880 and SD1302.

Supplementary Figure S8

(A and B) Inhibitory effect of hnRNP D on HPV16 E6 or E1 mRNA translation was demonstrated by in vitro transcription and translation assay in the presence or absence of recombinant hnRNP D protein. T7 promoter driven tagged-HPV16 E6 expressing plasmid (A) or FLAG-E1 expressing plasmid (B) was added in vitro transcription and in vitro translation reaction mixture in the absence (-) or presence (+) of

recombinant hnRNP D protein or BSA. Resulted proteins were analyzed by Western-blotting using anti-E6 antibody (A) or anti-FLAG antibody (B). Splice donor sites of HPV16 E6 and E1 were mutated as pXH856SDmF or p16E1SDm-3xF, respectively. HPV16 E6 protein was fused with TrxA-6xHis-S-Tag-HA tag at the N-terminus to increase the molecular weight (predicted size is 36 kD), separating from the unspecific bands detected in Western-blotting. **(C)** Effect of hnRNP D on translation of luciferase control mRNA was analysed by luciferase assay. Instead of E6 or E1 expressing plasmid, luciferase expressing plasmid was used. **(D)** The protein amount of recombinant hnRNP D and BSA used for the reaction indicated in (A) and (B) was confirmed by Coomassie staining (final 0.5uM of proteins in a reaction). **(E)** The quality and identity of recombinant hnRNP D protein was confirmed by Western-blotting using anti-hnRNP D antibody.

Supplementary Figure S1

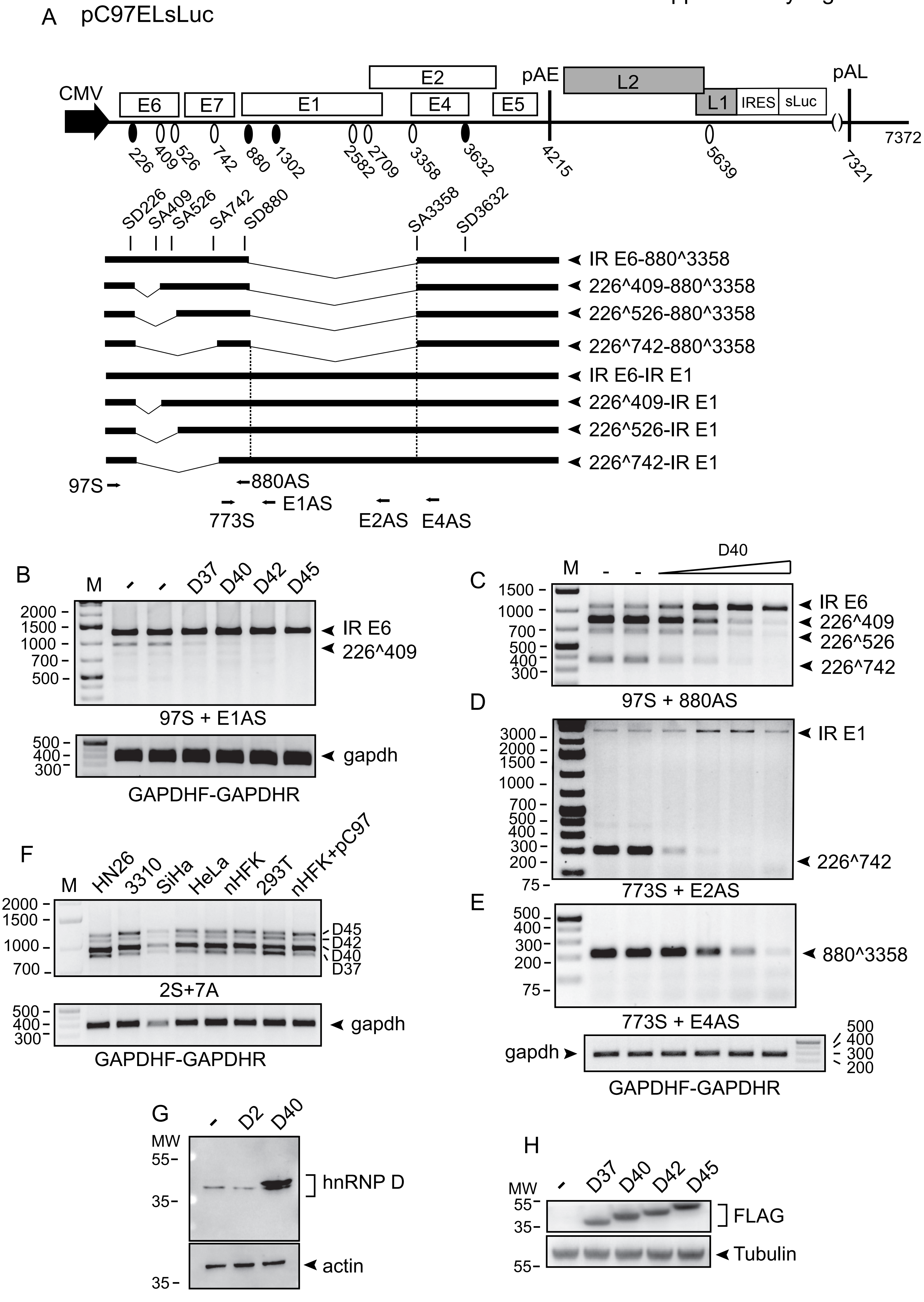

Supplementary Figure S2

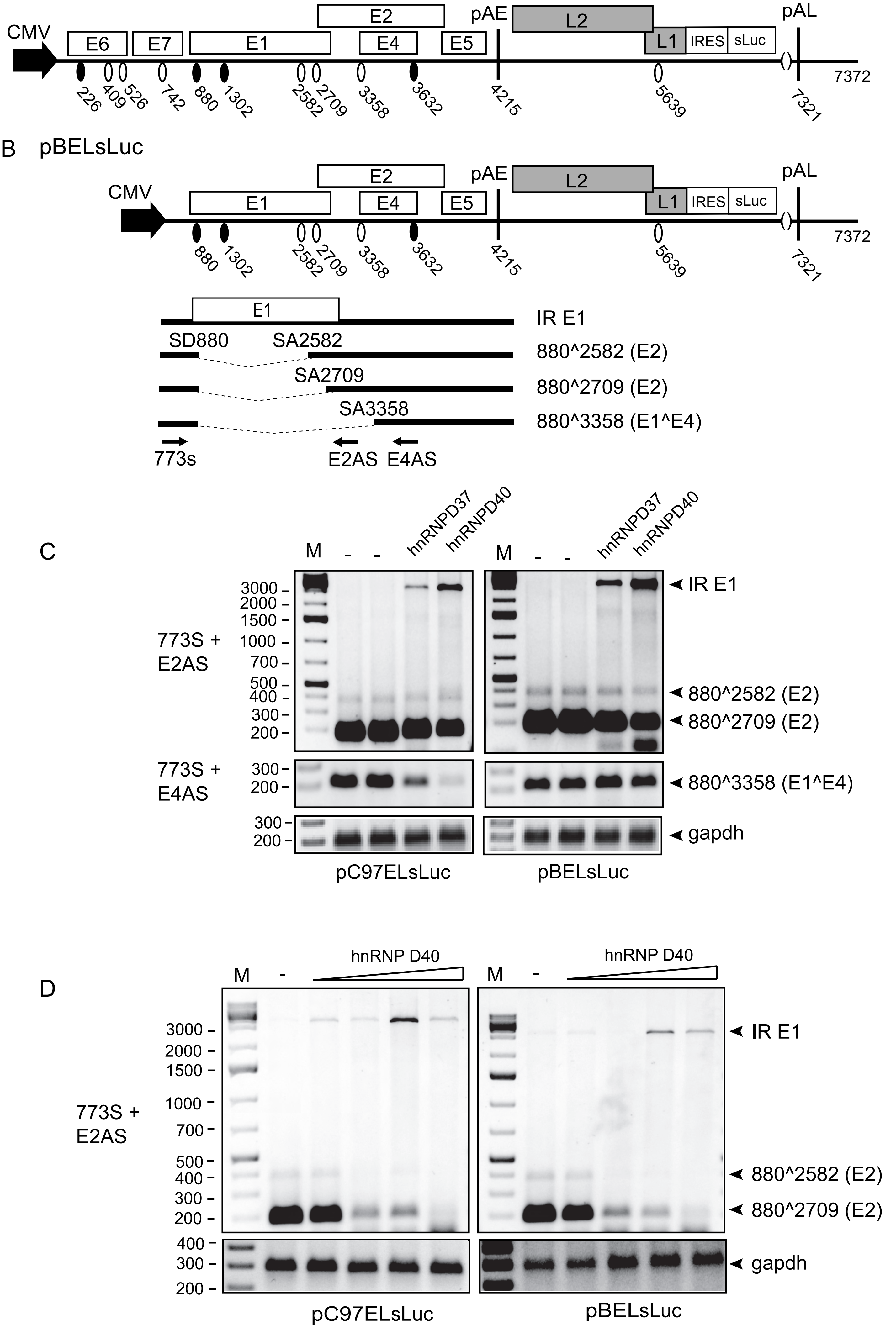

Supplementary Figure S3

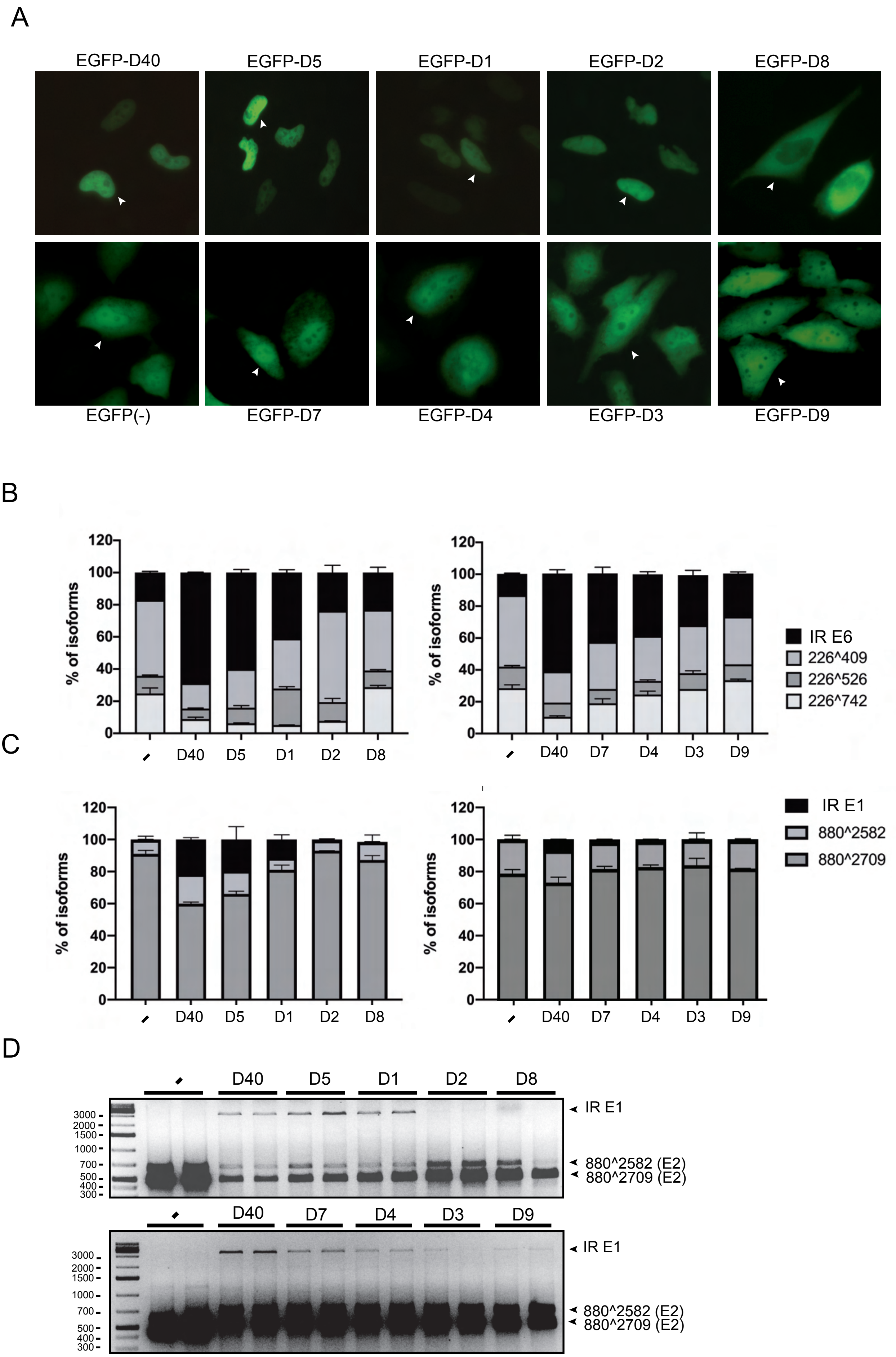

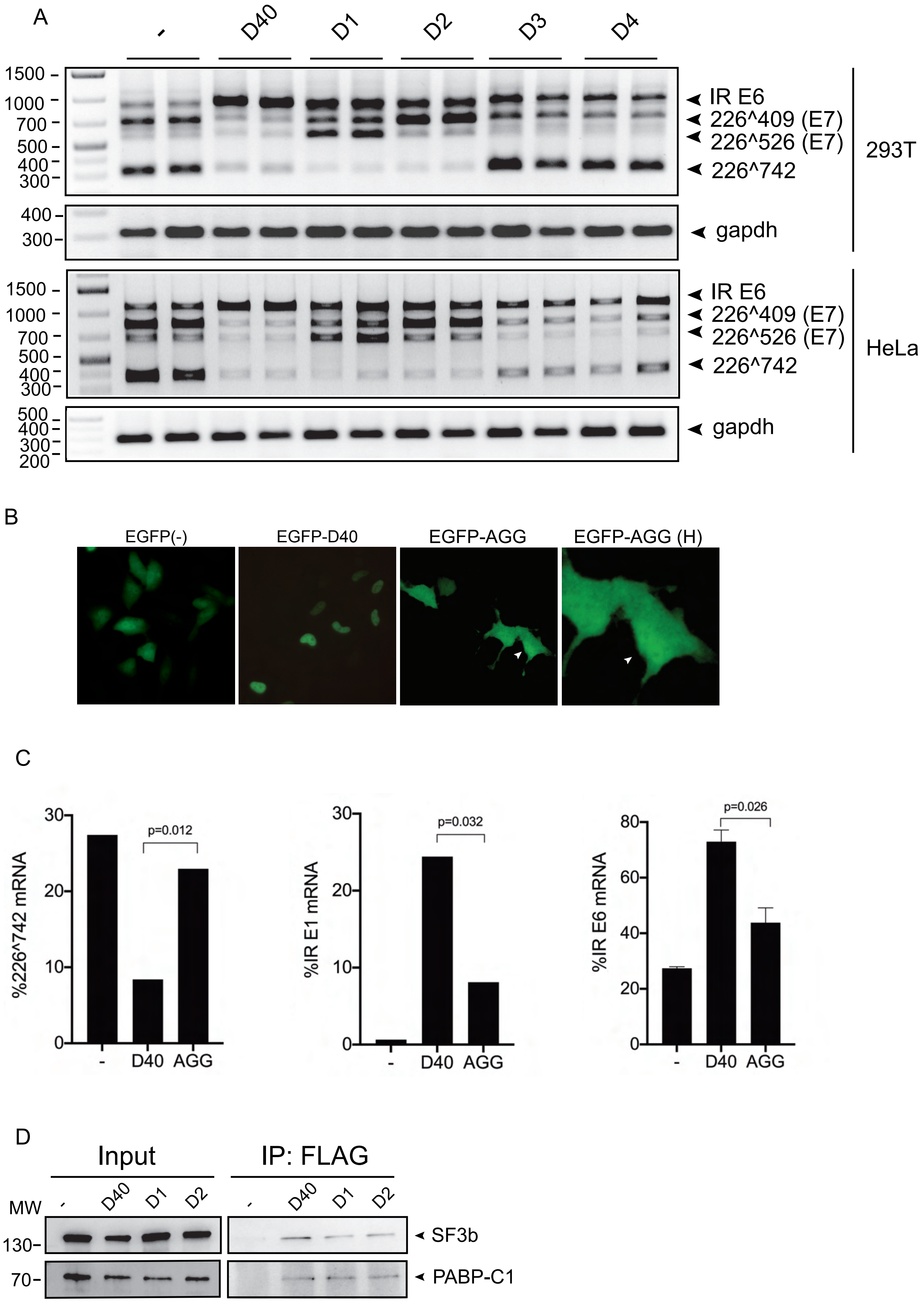
A
-
D40
D1
D2
D3
D4
1500
1000
700
500
400
300
IR E6
226^409 (E7)
226^526 (E7)
226^742
gapdh
293T
HeLa
B
EGFP(-)
EGFP-D40
EGFP-AGG
EGFP-AGG (H)
C
%226^742 mRNA
%IR E1 mRNA
%IR E6 mRNA
p=0.012
p=0.032
p=0.026
AGG
D
Input
IP: FLAG
MW
130
70
SF3b
PABP-C1

Supplementary Figure S5

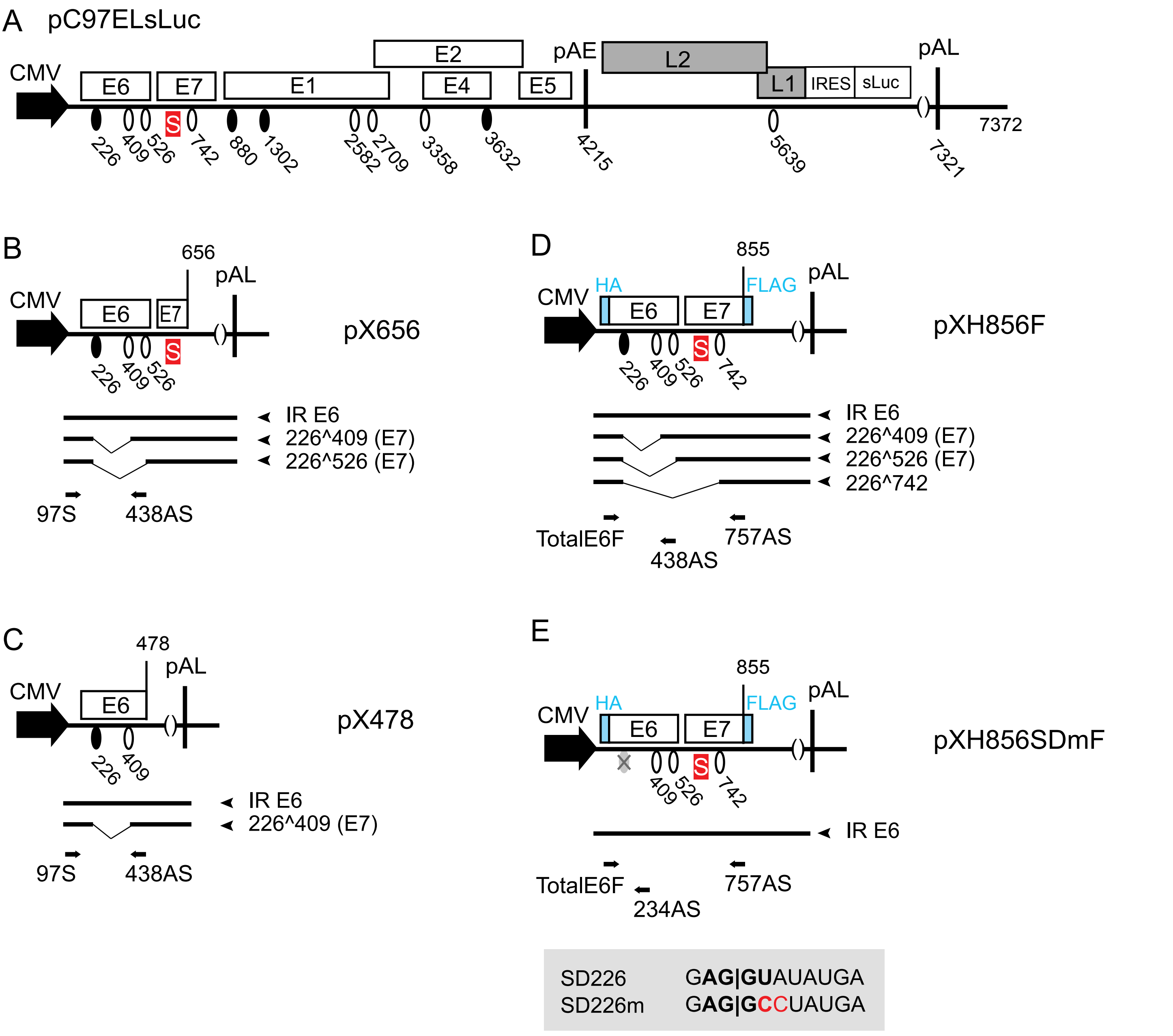

Supplementary Figure S6

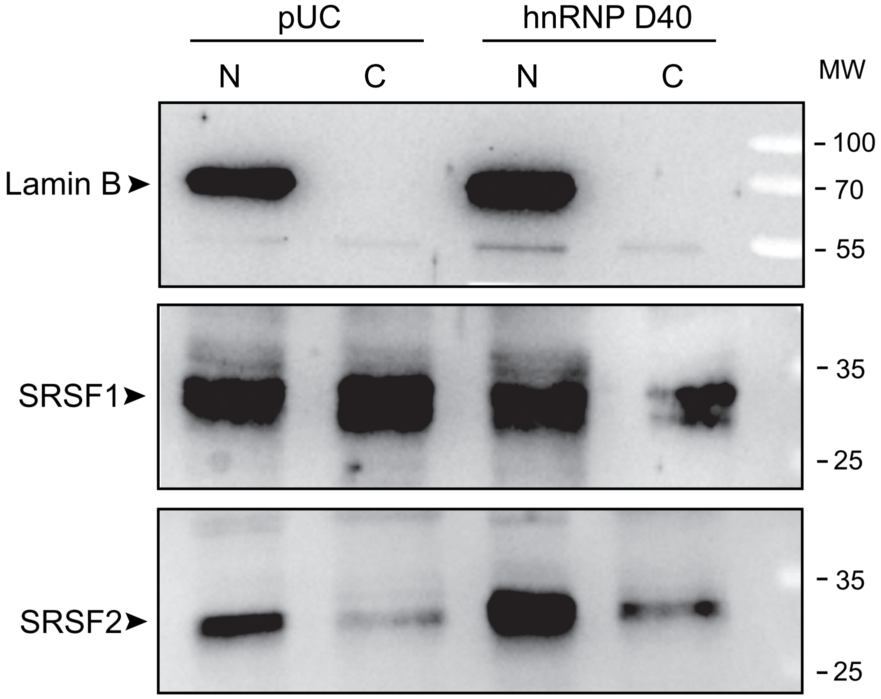

Supplementary Figure S7

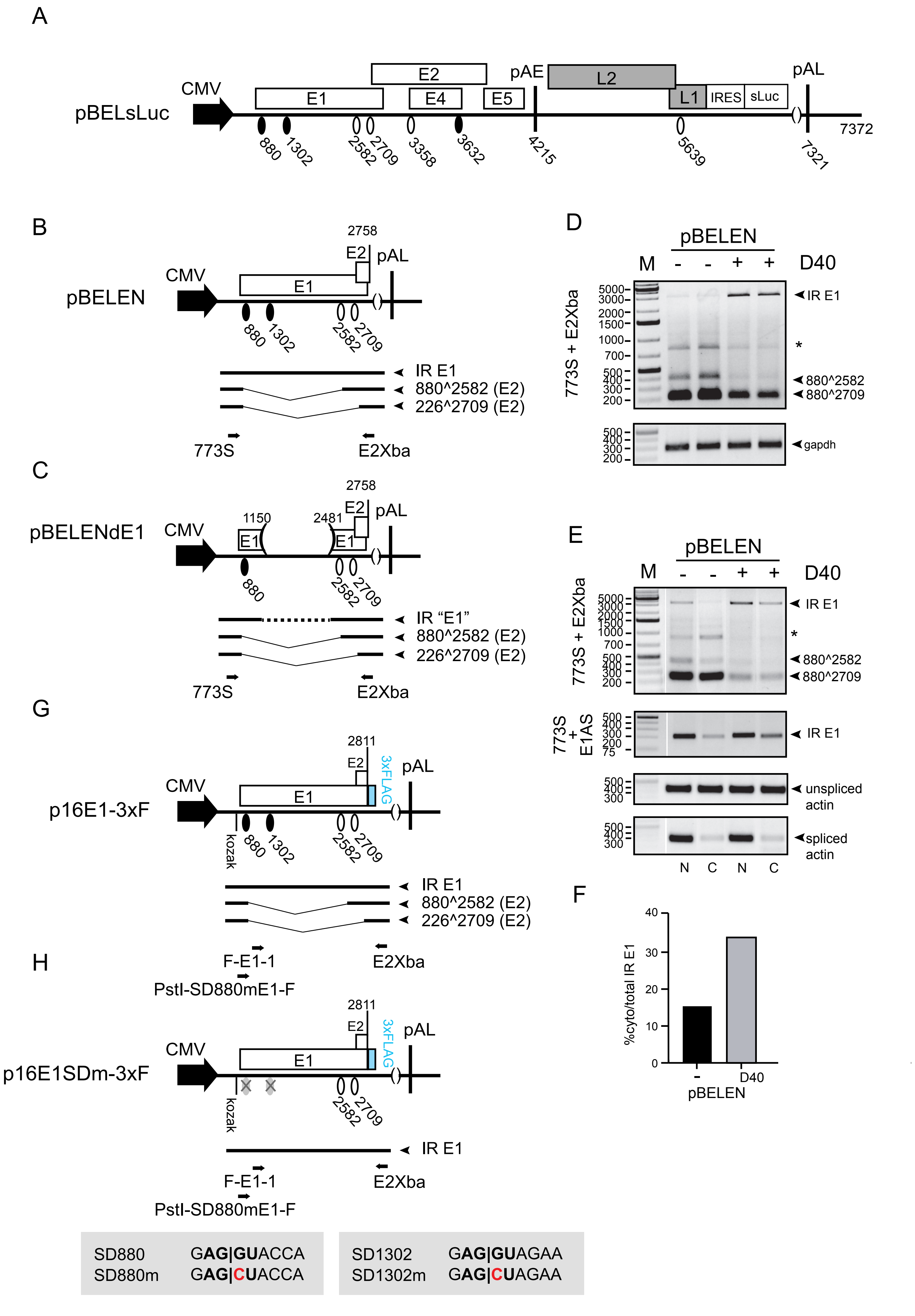

Supplementary Figure S8

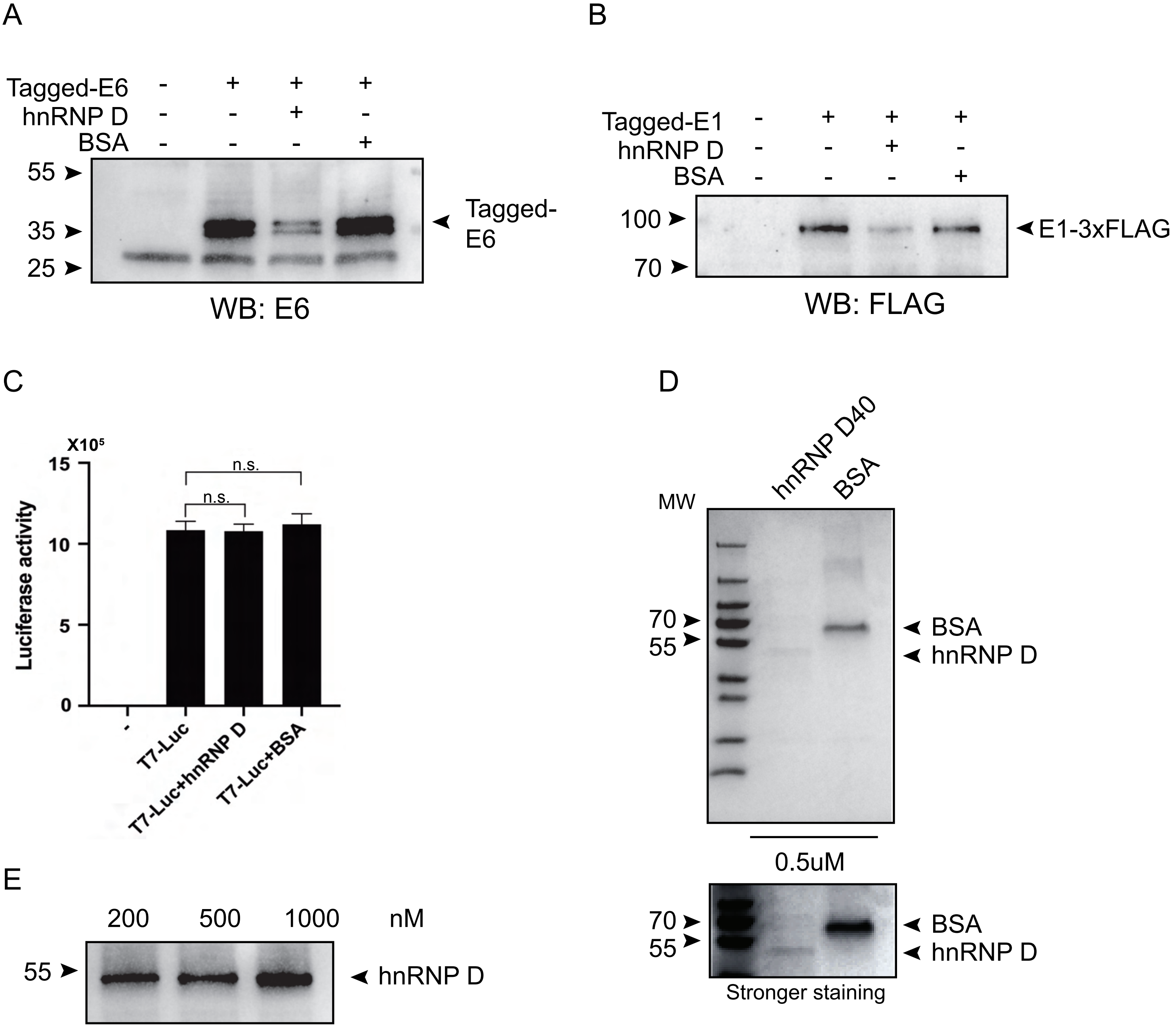

Supplementary Table 1. Sequences of PCR primers

Amplified region	primers	Sequence 5'-3'
E6/E7	97S	GTCGACCTGCAATGTTTCAGGACCC
	880AS	GAAACCATAATCTACCATGGCTGATC
	438AS	GCTCGAGGGAATCTTTGCTTTTTGTCCAGATGTCT
	757AS	CGTGTGTGCTTTGTACGCACAACCG
	TotalE6F	TGTTTCAGGACCCACAGGAG
	TotalE6R	TTGCTTGCAGTACACACATTC
	234AS	TGCATAAATCCCGAAAAGCAAAGT
E2	773S	GCACACACGTAGACATTCGTACTTTG
	E2AS	GTCCAGATTAAGTTTGCACGAGGAC
	E2QAS	CAGCCAGCGTTGGCACCACCT
E4	773S	GCACACACGTAGACATTCGTACTTTG
	E4AS	CCTCTCCTGAAATTATTAGGCAGCA
E1	773S	GCACACACGTAGACATTCGTACTTTG
	E1AS	CCATCCATTACATCCCGTACC
	1302S	CTGAAGTGGAAACTCAGCAGATGTTAC
	2293AS	TAATGATTTACCTGTGTTAGCTGCACC
	880S	GAAACCATAATCTACCATGGCTGATC
	F-E1-1	AGTAGAGCTGCAAAAAGGAGATTA
	PstI-SD880mE1-F	GATCCTGCAGCTACCAATGGGGAAGAGGGTAC
	E2Xba	AACCAGGTTCTAGACAAACTTAATCTGGACCACG
LoxP	16S	TATGTATGGTATAATAAACACGTGTGTATGTG
	16A	GCAGTGCAGGTCAGGAAAACAGGGATTTGGC
hnRNPD isoforms	2S	GAAGATTGACGCCAGTAAGAACG
	7A	TACCTTCCCATAACCACTCTGCT
pX478	B97S	GCGCGCTGCAATGTTTCAGGACCC
	X478A	GCTCGAGGGAATCTTTGCTTTTTGTCCAGATGTCT
pX656	B97S	GCGCGCTGCAATGTTTCAGGACCC
	X656A	CCTCGAGCTGTCATTTAATTGCTCATAACAG
pXH856F	sense	GACAGCGCGCACCATGTACCCATACGATGTTCCAGATTACGCTATGTTTCAGGACCCACAG
	anti sense	TTACTCGAGCTACTTATCGTCGTCATCCTTGTAATCTGGTTTCTGAGAACAGAT
pXH856SDmF	sense	CTGCGACGTGAGGCCTATGACTTTGC
	anti sense	GCAAAGTCATAGGCCTCACGTCGCAG
p16E1-3xF	sense	ATCAGCGCGCCACCATGGCTGATCCTGCAGGTAC
	anti sense	TAATCGATGTCATGATCTTTATAATCACCGTCATGGTCTTTGTAGTCTAATGTGTTAGTATTTTG
	anti sense	TCCCCTCGAGCTACTTGTCATCGTCATCCTTGTAATCGATGTCATGATC
p16E1SDm-3xF	sense	GATCCTGCAGCTACCAATGGGGAAGAGGGTAC
	anti sense	GGCGCCCTTCTAGCTGTAACATCTGCTGAGT
	anti sense	TACAGCTAGAAGGGCGCCATGAGACTG
pflag-D40	sense	GTTGCGCGCGTTATCATGGACTACAAAGACGATGACGACAAG
	anti sense	CCTCGAGTTAGTATGGTTTGTAGCTATTTTGATGACCACC
pD1,pD1-AGG and pD1-Q6A	sense	GGCGCGCGCCACCATGGACTACAAAGACGATGACGACAAGATGTTTATAGGAGGCCTTAGCTGGG
	anti sense	CCTCGAGTTAGTATGGTTTGTAGCTATTTTGATGACCACC
pD2,pD2-AGG and pD2-Q6A	sense	GGCGCGCGCCACCATGGACTACAAAGACGATGACGACAAGATGGTTAAAAAAATTTTTGTTGGTGGCC
	anti sense	CCTCGAGTTAGTATGGTTTGTAGCTATTTTGATGACCACC
pD5	sense	GTTGCGCGCGTTATCATGGACTACAAAGACGATGACGACAAGGGAAGCGGAGCCGGGAC
	anti sense	CCTCGAGTTAGTATGGTTTGTAGCTATTTTGATGACCACC
pD3	sense	GTTGCGCGCGCCACCATGGACTACAAAGACGATGACGACAAG
	anti sense	CCGCTCGAGTTAGGCTTTGGCCCTTTTAGGATC
pD4	sense	GTTGCGCGCGCCACCATGGACTACAAAGACGATGACGACAAG
	anti sense	CCGCTCGAGTTACATGGCTACTTTTATTTCACATTTAC
pD7	sense	GTTGCGCGCGCCACCATGGACTACAAAGACGATGACGACAAG
	anti sense	CTTCTCGAGTTAAGATCCCCACTGTTGCTGTTGCTGATATTG
pD8	sense	GGCGCGCGCCACCATGGACTACAAAGACGATGACGACAAGATGTTTATAGGAGGCCTTAGCTGGG
	anti sense	CCGCTCGAGTTACATGGCTACTTTTATTTCACATTTAC
pEGFP-D40	sense	GTTAAGCTTGTTATCATGGACTACAAAGACGATGACGACAAG
	anti sense	CTTGGATCCTTAGTATGGTTTGTAGCTATTTTGATGACCACC
pEGFP-D1	sense	GTTAAGCTTGTTATCATGGACTACAAAGACGATGACGACAAG
	anti sense	CTTGGATCCTTAGTATGGTTTGTAGCTATTTTGATGACCACC
pEGFP-D2	sense	GTTAAGCTTGTTATCATGGACTACAAAGACGATGACGACAAG
	anti sense	CTTGGATCCTTAGTATGGTTTGTAGCTATTTTGATGACCACC
pEGFP-D5	sense	GTTAAGCTTGTTATCATGGACTACAAAGACGATGACGACAAG
	anti sense	CTTGGATCCTTAGTATGGTTTGTAGCTATTTTGATGACCACC
pEGFP-D3	sense	GTTAAGCTTGTTATCATGGACTACAAAGACGATGACGACAAG
	anti sense	CTTGGATCCTTAGGCTTTGGCCCTTTTAGGATC
pEGFP-D4	sense	GTTAAGCTTGTTATCATGGACTACAAAGACGATGACGACAAG
	anti sense	CTTGGATCCTTACATGGCTACTTTTATTTCACATTTAC
pEGFP-D7	sense	GTTAAGCTTGTTATCATGGACTACAAAGACGATGACGACAAG
	anti sense	CTTGGATCCTTAAGATCCCCACTGTTGCTGTTGCTGATATTG
pEGFP-D8	sense	GTTAAGCTTGTTATCATGGACTACAAAGACGATGACGACAAG
	anti sense	CTTGGATCCTTACATGGCTACTTTTATTTCACATTTAC

pEGFP-D9	sense	GTTAAGCTTGTTATCATGGACTACAAAGACGATGACGACAAG
	anti sense	CTTGGATCCTTACCTCCTATAAACATTTTCC
GAPDH	GAPDHF	ACCCAGAAGACTGTGGATGG
	GAPDHR	TTCTAGACGGCAGGTCAGGT
spliced actin	actin-s	TGAGCTGCG TGTGGCTCC
	actin-a	GGCATGGGGGAGGGCATACC
unspliced actin	actin-s1	CCAGT GGCTTCCCCAGTG
	actin-a	GGCATGGGGGAGGGCATACC
Involucrin	INV_S	CTCTGCCTCAGCCTTACTGTGA
	INV_R	GCTCCTGATGGGTATTGACTGG
pET32a-HA-E6SDm	sense	TATGAATTCATGTACCCATACGATGTTCCAGAT
	anti sense	TTACTCGAGCTATGGTTTCTGAGAACAGATGGGG

Supplementary Table 2. Antibodies used in WB, CLIP, RIP and Co-IP

	Cat.	Company
AUF1/hnRNPD	12382S	Cell Signaling
hnRNPM	ab177957	abcam
hnRNPU	ab10297	abcam
Flag,M2	F3165/F1804	Sigma Aldrich
U1 70K	ab83306	abcam
U2AF65	sc-53942	Santa Cruz
U2AF35	ab172614	abcam
HPV16 E6	GTX132686	Genetex
HPV16 E7	GTX133411	Genetex
β-actin	A5441	Sigma Aldrich
Tubulin	T9026	Sigma Aldrich
Histone	4620S	CST
involucrin	sc-21748	Santa Cruz
HA	sc-7392	Santa Cruz
Lamin B	ab16048	abcam
SRSF1	ab38017	abcam
SRSF2	ab204916	abcam
SF3b	ab172634	abcam
PABP-C1	ab6125	abcam
veriblot	ab131366	abcam
Normal Rabbit IgG	2729S	Cell Signaling
Normal Mouse IgG	12-371	Millipore
anti-Mouse IgG-HRP	A9044	Sigma Aldrich
anti-Rabbit IgG-HRP	A9169	Sigma Aldrich

Supplementary Table 3. Sequences of oligos for pull down assay

HPV16 pull down region	Sequence 5'-3'
1, 178-214	UAUAAUAUUAGAAUGUGUGUACUGCAAGCAACAGUUA
2, 196-233	AGUUACUGCGACGUGAGGUAUAUGACUUUGCUUUUCGG
3, 229-266	UUCGGGAUUUAUGCAUAGUAUAUAGAGAUGGGAAUCCA
4, 276-313	AUCCAUAUGCUGUAUGUGAUAAAUGUUUAAAGUUUUAU
5, 309-346	UUUAUUCUAAAAUUAGUGAGUAUAGACAUUAUUGUUAU
6, 342-379	GUUAUAGUUUGUAUGGAACAACAUUAGAACAGCAAUAC
7, 375-412	AAUACAACAAACCGUUGUGUGAUUUGUUAAUUAGGUGU
8, 408-445	GGUGUAUUAACUGUCAAAAGCCACUGUGUCCUGAAGAA
9, 441-478	AAGAAAAGCAAAGACAUCUGGACAAAAAGCAAAGAUUC
10, 474-498	GAUUCCAUAAUAUAAGGGGUCGGUG
11, 492-516	GUCGGUGGACCGGUCGAUGUAUGUC
12, 506-530	CGAUGUAUGUCUUGUUGCAGAUCAU
13, 521-545	UGCAGAUCAUCAAGAACACGUAGAG
14, 536-560	ACACGUAGAGAAACCCAGCUGUAAU
15, 549-573	CCCAGCUGUAAUCAUGCAUGGAGAU
16, 564-588	GCAUGGAGAUACACCUACAUUGCAU
17, 579-604	UACAUUGCAUGAAUAUAUGUUAGAUU
18, 594-620	UAUGUUAGAUUUGCAACCAGAGACAAC
19, 611-635	CAGAGACAACUGAUCUCUACUGUUA
20, 631-665	UGUUAUGAGCAAUUAAAUGACAGCUCAGAGGAGGA
21, 661-695	GAGGAGGAUGAAAUAGAUGGUCCAGCUGGACAAGC
22, 691-725	CAAGCAGAACCGGACAGAGCCCAUUACAAUAUUGU
23, 721-755	AUUGUAACCUUUUGUUGCAAGUGUGACUCUACGCU
24, 751-785	ACGCUUCGGUUGUGCGUACAAAGCACACACGUAGA
25, 781-815	GUAGACAUUCGUACUUUGGAAGACCUGUUAAUGGG
26, 811-845	AUGGGCACACUAGGAAUUGUGUGCCCCAUCUGUUC
27, 841-875	UGUUCUCAGAAACCAUAAUCUACCAUGGCUGAUCC
28, BSD+GGG	CUGAUCCUGCAGGUACCAAUGGGGAAGAGGGUACGGGAUGUA

Supplementary Table 4. Primer pair information

RT-PCR				
Amplified region	Sense primer	Antisense primer	Primer location	Shown in following Figures:
E6/E7	97S	880AS	Fig. 1C	Fig. 2A, 3B, 4D, 4F, 5B, 5I, 6C, 8B, 8F, 8K
	97S	438AS	Fig. 1C, 5L, S5B, S5C	Fig. 5L, 6C, 6I, 6M, 8I, 8J
	TotalE6F	Total E6R	Fig. 1C, Fig 5L	Fig. 5L
	TotalE6F	757AS	Fig. 1C, S5D, S5E	Fig. 6C
E1/E2	773S	E2AS	Fig. 1E, 3E	Fig. 2A, 3C, 3F, 7A, 8D, 8G
	773S	E2QAS	Fig. 1E	Fig. 4E, 4G, 5C, 5J
	773S	E2Xba	Fig. 1E, S7C	Fig. 7D
	F-E1-1	E2Xba	Fig. 1E, S7H	Fig. 7G
	PstI-SD880mE1-F	E2Xba	Fig. 1E, S7H	Fig. 7G
	1302S	2293AS	Fig. 1E, 3E	Fig. 3G
	773S	E1AS	Fig. 1E, 3E	Fig. 3H, 7J, 8E, 8K, 8L
E4(880^3358)	773S	E4AS	Fig. 1C	Fig. 2B, 8C, 8I

RT-qPCR				
Amplified region	Sense primer	Antisense primer	Primer location	Shown in following Figures:
Spliced E2 mRNA	773S	E2AS	Fig. 1E, 3E	Fig. 3D, 3J
IR E1 mRNA	773S	E1AS	Fig. 1E, 3E	Fig. 3D, 3J
IR E6 mRNA	TotalE6F	234AS	Fig. 1C, Fig S5	Fig. 6H
